# Synthesis, computational docking and molecular dynamics studies of a new class of spiroquinoxalinopyrrolidine embedded chromanone hybrids as potent anti-cholinesterase agents†

**DOI:** 10.1039/d4ra02432j

**Published:** 2024-06-12

**Authors:** Natarajan Arumugam, Datta Darshan V. M., Vishal Venketesh, Sai Sanwid Pradhan, Anuj Garg, Venketesh Sivaramakrishnan, Subbarao Kanchi, Sakkarapalayam M. Mahalingam

**Affiliations:** a Department of Chemistry, College of Science, King Saud University P. O. Box 2455 Riyadh 11451 Saudi Arabia anatarajan@ksu.edu.sa mahasmole@gmail.com; b Disease Biology Lab, Department of Biosciences, Sri Sathya Sai Institute of Higher Learning, Prasanthi Nilayam Andhra Pradesh 515134 India; c Department of Physics, Sri Sathya Sai Institute of Higher Learning, Prasanthi Nilayam Andhra Pradesh 515134 India; d Department of Chemistry, Purdue University 720 Clinic Drive West Lafayette Indiana 47907 USA

## Abstract

Novel structurally intriguing heterocycles embedded with spiropyrrolidine, quinoxaline and chromanone units were synthesized in good yields using a [Bmim]Br accelerated multicomponent reaction strategy. The key step of the reaction is 1,3-dipolar cycloaddition involving highly functionalized dipolarophile, *viz.* 3-benzylidenechroman-4-one, to afford spiroquinoxalinopyrrolidine embedded chromanone hybrid heterocycles. The formation of spiro products occurs *via* two C–C, two N–C and one C–N bonds possessing four adjoining stereogenic centers, two of which are spiro carbons. The newly synthesized spiro compounds showed potent acetylcholinesterase and butyrylcholinesterase inhibitory activities. Moreover, compounds with fluorine displayed the highest AChE (3.20 ± 0.16 μM) and BChE (18.14 ± 0.06 μM) inhibitory activities. Further, docking studies, followed by all-atom molecular dynamics, showed results that are consistent with *in vitro* experimental findings. Although docking scores for the synthesized derivatives were higher than those of the standard drug, MD MMPBSA results showed better binding of synthesized derivatives (−93.5 ± 11.9 kcal mol^−1^) compared to the standard drug galantamine (−66.2 ± 12.3 kcal mol^−1^) for AChE but exhibited similar values (−98.1 ± 11.2 and −97.9 ± 11.5 kcal mol^−1^) for BChE. These differences observed in drug binding with AChE/BChE are consistent with RMSD, RMSF, LIG plots, and FEL structural analysis. Taken together, these derivatives could be potential candidates as inhibitors of AChE and BChE.

## Abstract 2

Spiropyrrolidines were synthesized in good yields by cycloaddition protocol, these compounds showed potent cholinesterase inhibitory activities. Experimental *in vitr* o findings are consistent with docking and molecular dynamics simulations.

## Graphical abstract

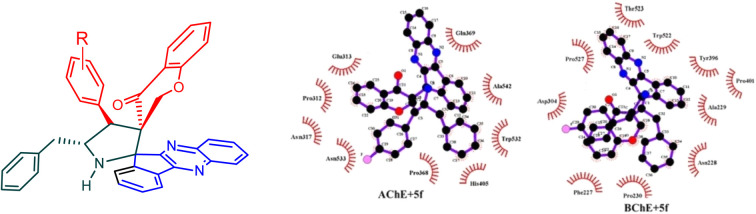

## 1. Introduction

Alzheimer's disease (AD), Parkinson's disease, and Huntington's disease (HD) are among the neurodegenerative diseases associated with protein aggregation and lead to cognitive, behavioral, and motor dysfunction.^1–5^ A study conducted by the WHO estimated that over thirty seven million individuals around the world are terribly affected by AD^6^ and that the number will reach 66 million by 2030 and 115 million by 2050.^7^ The disease is clinically identified by amnestic cognitive impairment, learning disability, memory loss, and a wide array of neuropsychiatric signs during midlife and late life, which indicate that this disease affects the ability to think and perform daily activities independently.^1,8,9^ The cholinergic hypothesis suggests that these patients have severe impairments in their cholinergic systems in the forebrain, cortex, and hippocampus. Ultimately, this leads to severe cognitive and memory impairments due to the diminished secretion and concentration of the acetylcholine (Ach) neurotransmitter in the synaptic cleft. However, AD is primarily connected with deficits in acetylcholine (ACh), amyloid peptide deposits, and hyperphosphorylated tau protein.^10,11^ Hence, cholinesterase inhibitors are used to maintain or prolong the effects of residual acetylcholine to treat AD.^12^

In the human brain, there are two-cholinesterase enzymes, acetylcholinesterase (AChE) and butyrylcholinesterase (BChE), which play an essential role in AD complications mainly owing to their presence in the peripheral and central nervous system and neuromuscular junction. In the body, acetylcholine is regulated and degraded by these enzymes and has been thoroughly examined as a target for AD treatment. Acetylcholinesterase has been extensively investigated between the two enzymes involved in cholinergic transmission and cholinnoceptive neurons.^13^ Cholinergic neurons, particularly acetylcholine (ACh), have a good connection with memory encoding, memory function and the recovery process.^14^ In patients with AD, Ach levels are decreased due to the AChE enzyme that produces choline and acetate, which is further connected with memory cognitive and memory impairments.^15,16^

The cholinesterase inhibitors are mainly based on enhancing cholinergic transmission at the cholinergic synapses found in the atomic nervous system.^17^ Many drugs in development target acetylcholinesterase, such as galantamine, donepezil, and rivastigmine, which are all used to correct neurotransmitter disorders or treat symptoms.^18^ Available drugs that inhibit AChE are known to have limitations *viz.* short duration of action, low bioavailability, high toxicity profile and narrow therapeutic effects. Therefore, it is imperative to discover and develop structurally new drug candidates with reduced toxicity and improved potency as AChE inhibitors.

**Fig. 1:**
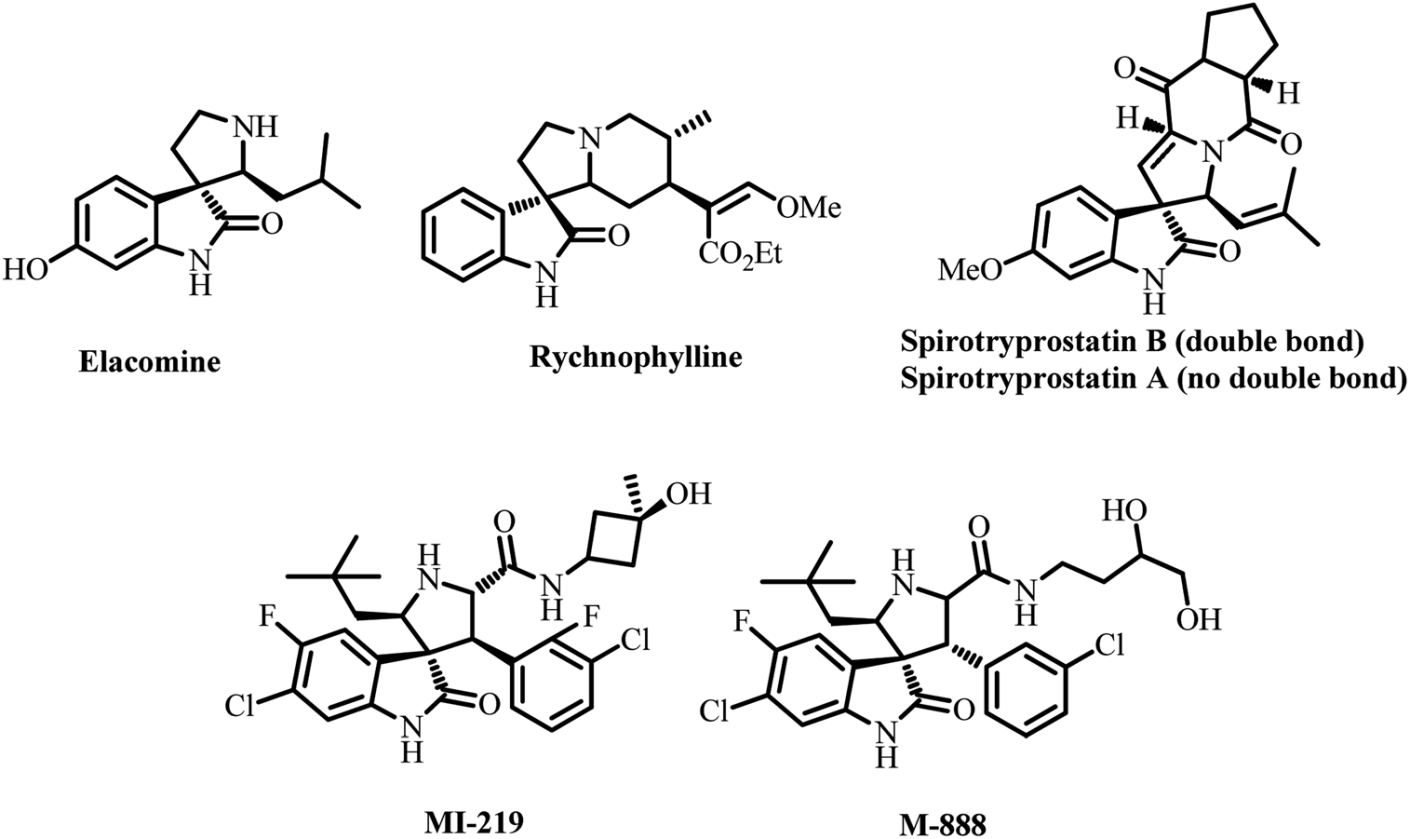
Biologically relevant natural and synthetic spiropyrrolidines.

Spiro heterocycles are attractive in this regard because spiro compounds are present as an active entity in numerous biologically active compounds.^19^ The spiropyrrolidine structural units are particularly abundant in naturally occurring products and biologically active synthetic compounds, including horsfiline, elacomine, rhynchophylline, and spirotryprotatins A and B. These analogs and many additional synthetic spiropyrrolidines (Fig. 1) have been reported to display significant anticancer, antimicrobial, antimycobacterial, anti-inflammatory, analgesic, local anesthetic and cholinesterase inhibition activities.^20–24^ Spiroheterocycles employing multicomponent cycloaddition reactions have been a major focus of our research team. Among them, most of the spiro analogues exhibited better AChE/BChE inhibitory activities, and few of them displayed excellent activity than reference standard drugs.^25–28^

Prompted by these findings, the biological importance of cholinesterase inhibitors and the specification of the enzyme active site, we employed molecular simulation techniques to design efficient inhibitors of AChE and BChE enzymes. In this context, herein, we explored the potential structurally intriguing new class of heterocycles comprising spiropyrrolidine, quinoxaline and chromanone synthesized in ionic liquid accelerated cycloaddition reaction protocol. Ionic liquids are known as eco-friendly solvents in many organic syntheses^29–31^ because of their unique properties, including low vapour pressure, high thermal and chemical stability, non-inflammability solvating capability, and recyclability, and they play twin roles as reactant and catalyst in organic synthesis. For this reason, their use in organic synthesis has emerged as an important facet of green chemistry. Furthermore, computational docking and molecular dynamics studies were employed to explore the plausible binding interactions of the most active compounds.

## 2. Results and discussion

### 2.1. Chemistry

**Scheme 1:**
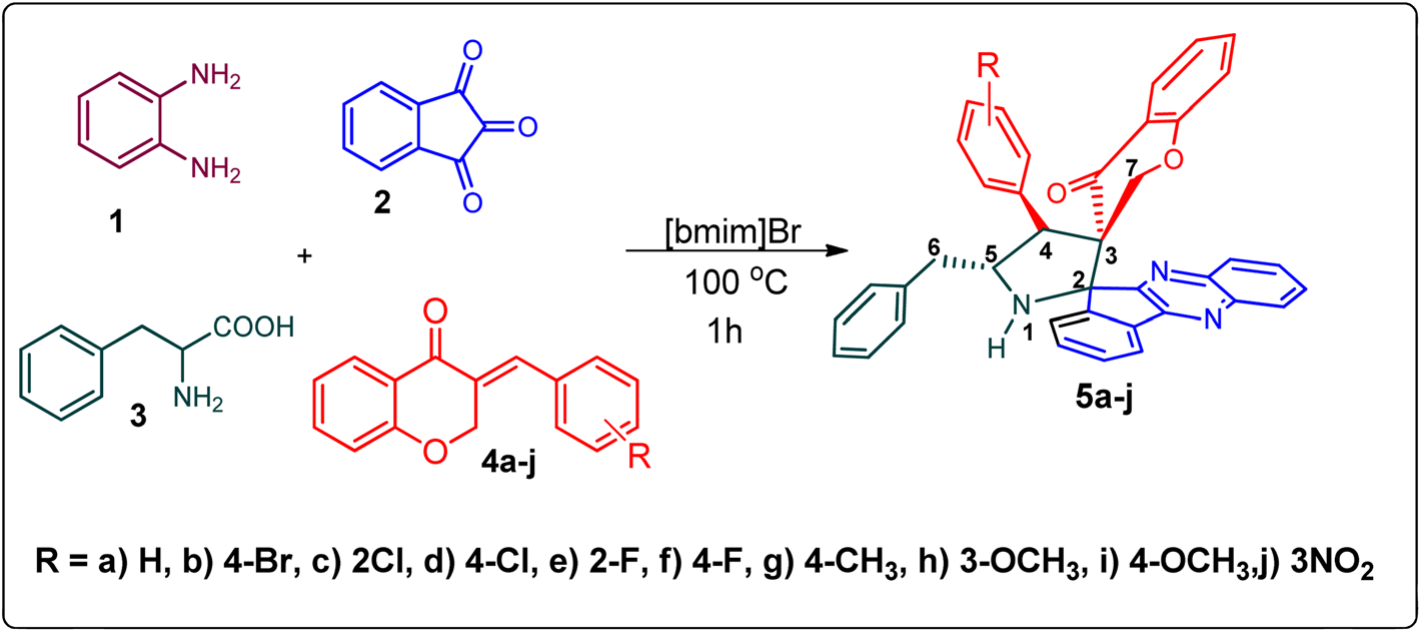
Synthesis of grafted chromanones 5a–j.

Various substituted dipolarophiles, *viz.* 3-(4-benzylidene) chroman-4-one 4a–j, were required for the preparation of spiroquinoxaline-embedded chromanones prepared from chromanone and various substituted arylaldehyde.^32^ Using starting substrates 4a–j, we performed our study on the cycloaddition tandem protocol of 3-(4-benzylidene)chroman-4-one 4 with a 1,3-dipole derived from quinoxalinone and amino acid heated to 100 °C in [Bmim]Br, resulting in the cycloadduct of spiroindenopyrrolidine grafted chromanone hybrids 5a–j as a sole product in good yields (Scheme 1). Initially, we performed a cycloaddition reaction with 3-(4-benzylidene) chroman-4-one (4c), *o*-phenylenediamine 1, ninhydrin 2, and l-phenylalanine 3 in MeOH under reflux for 3 h until the starting substrate was completely consumed. As an alternative to the green synthetic protocol, the cycloaddition reaction was performed with [Bmim]Br as a green solvent at 100 °C for one hour, and TCL confirmed the absence of reactants and the formation of the product. After dilution with EtOAc and H_2_O, ethyl acetate was concentrated at a reduced temperature. In the end, the residue was washed with Et_2_O to yield 92% pure product 5c.

**Fig. 2:**
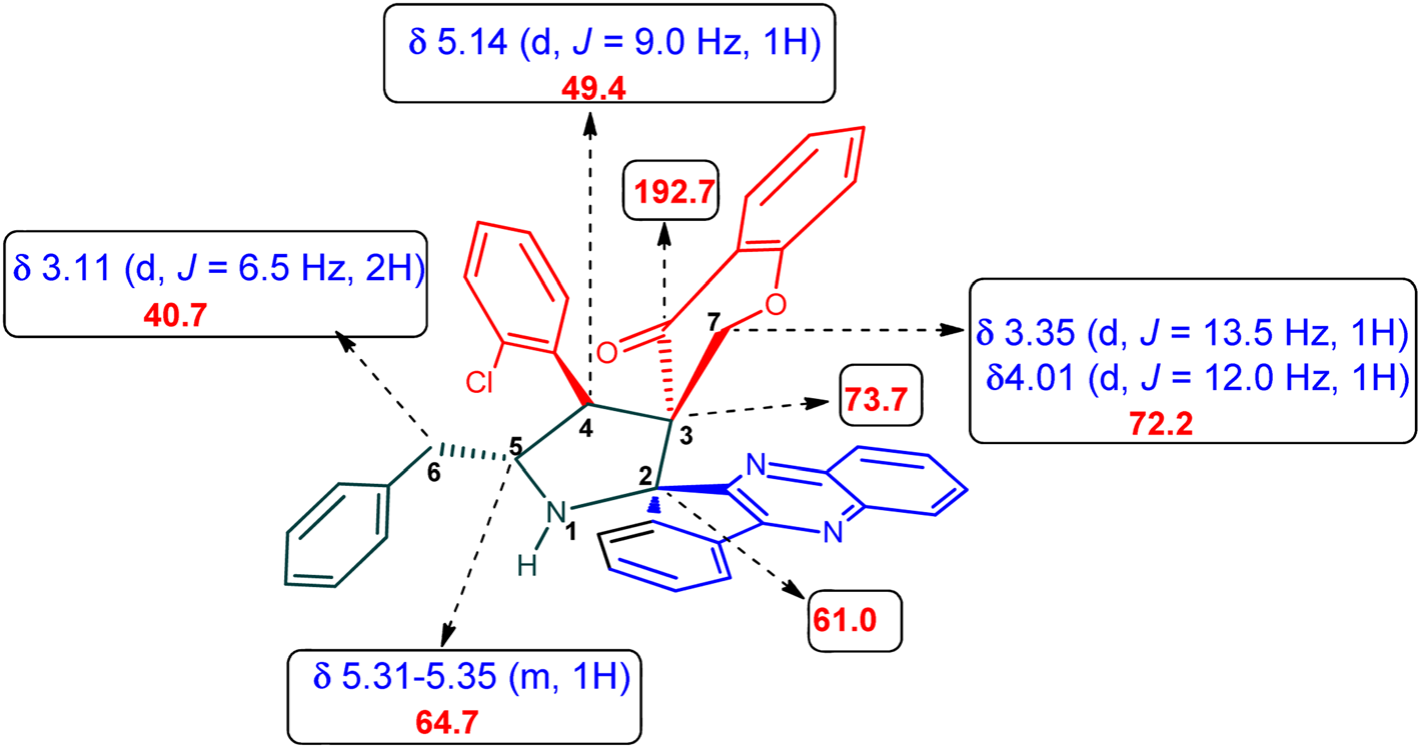
Selected chemical shift of 5c.

**Fig. 3:**
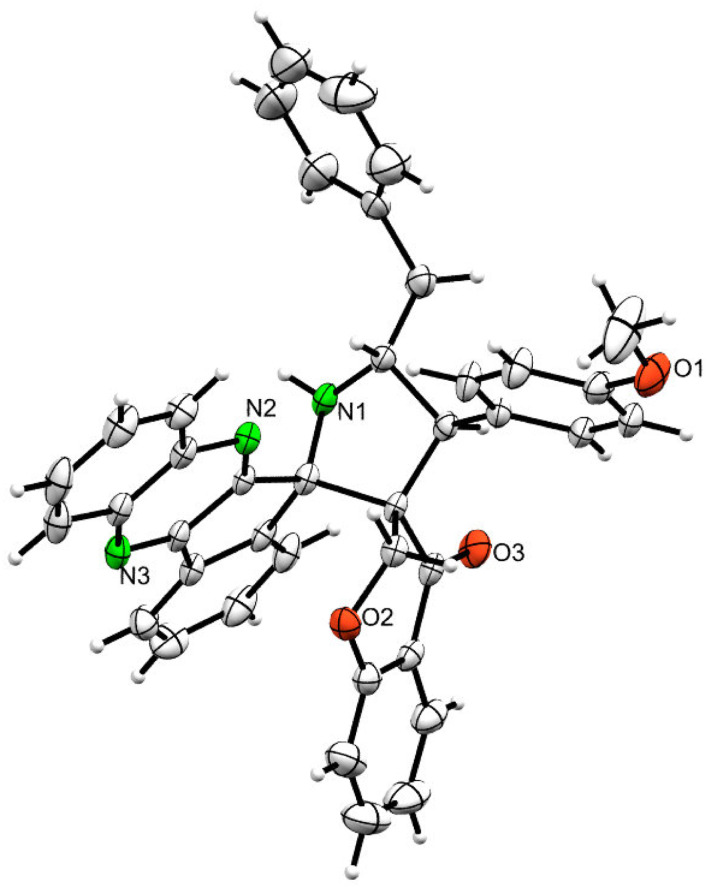
ORTEP diagram of 5i.

The ^1^H NMR spectrum of 5c has a multiplet at *δ* 5.31–5.35 ppm assigned to H-5 hydrogen of the pyrrolidine ring, which showed (i) H, H–COSY correlations at two doublets at 5.14 ppm (*J* = 9.0 Hz) and *δ* 3.11 ppm (*J* = 6.5 Hz), corresponding to H-4 and H-6 hydrogens (Fig. 2). The chromanone methylene hydrogens (H-7) were shown as two doublets at *δ* 3.35 (*J* = 13.5 Hz) and 4.01 (*J* = 12.0 Hz). From the HMQC spectrum, the hydrogen signals correlate well with their carbon signals at *δ* 49.4, 40.7, 64.7 and 72.2, which correspond to C-4, C-6, C-5 and C-7, respectively. The signal at *δ* 192.7 ppm was assigned to chromanone carbonyl carbon. Further, the spiro carbon, methyl and methine carbon were undoubtably determined with no doubt through the DEPT-135 spectrum (*vide* ESI, Fig. S1–S5†). A single-crystal X-ray study (Fig. 3) of 5i unequivocally determined its regio- and stereochemistry.^33^

**Scheme 2:**
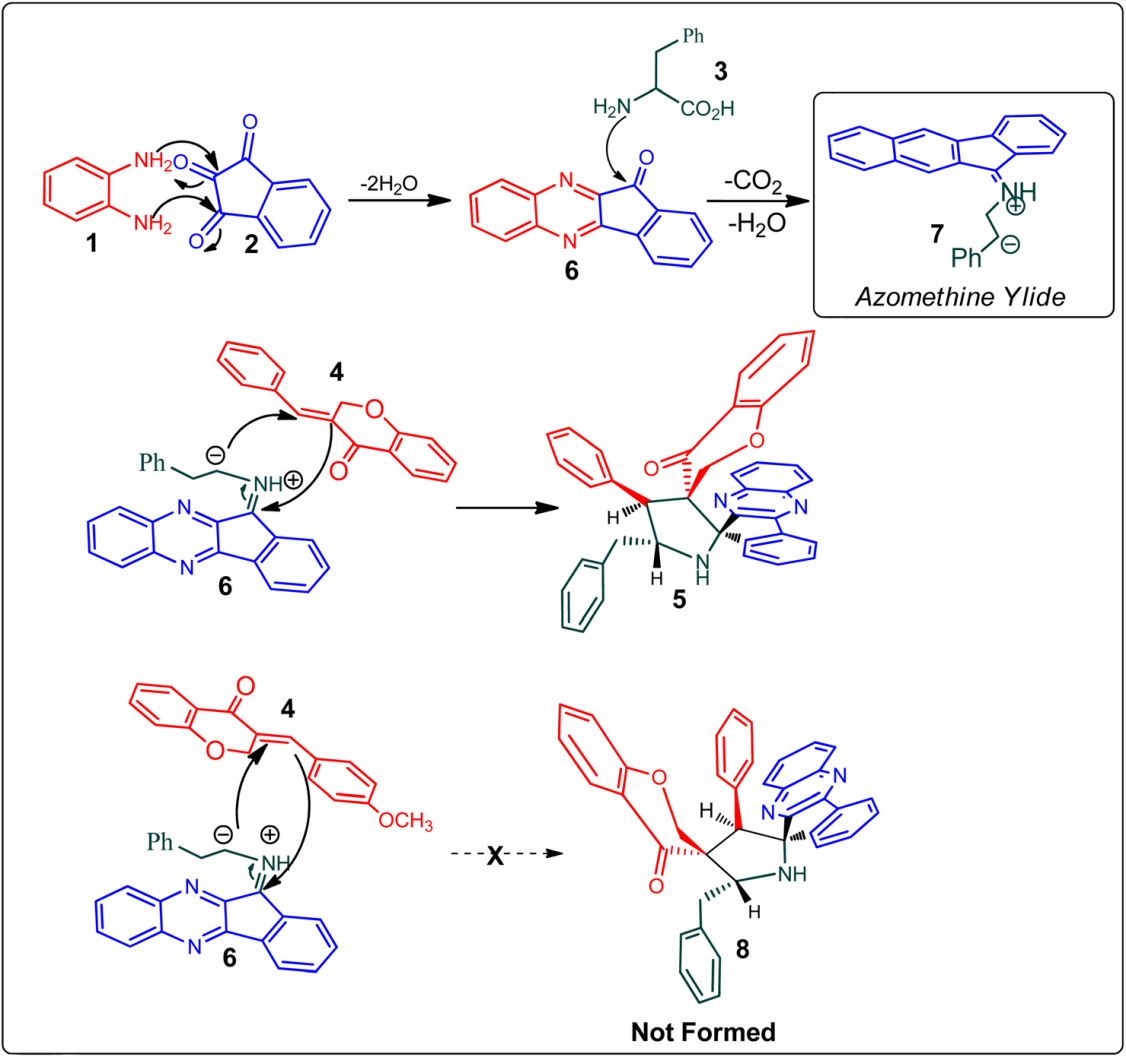
Mechanistic pathway for the formation of dispiropyrrolidine 5.

Scheme 2 demonstrates a plausible mechanism for the construction of the spirocompound-grafted chromanone 5. Initially, the reaction between 2 and 1 affords compound 6 by eliminating two moles of water, which then reacts with amino acid 3 to produce the non-stabilized ylide 7. Subsequently, ylide 7 was trapped in chromanone dipolarophile 4 furnishing 5 in preference over product 8, which was not obtained because the electron rich carbon of the 7 favorably reacts with electron deficient β-carbon of ketone 4. During a single synthetic transformation, two C–C, one C–N, and two N=C bonds are formed to produce the target compound 5.

### 2.2. Cholinesterase inhibitory activity

Acetylcholine is an important neurotransmitter involved in memory and cognition.^34^ The enzymes AChE and BChE act on these neurotransmitters and degrade them to attenuate acetylcholine-mediated effects.^35^ In neurodegenerative diseases, a reduced acetyl choline level is detrimental, leading to impaired memory and cognition.^36^ To improve the outcome and management of the disease, it is imperative to identify compounds with improved inhibitory potential and lower toxicity. In this context, the new class of spiroquinoxalinopyrrolidine embedded chromanones 5a–j was assayed owing to their *in vitro* cholinesterase inhibitory activity against AChE and BChE enzymes, and the results are shown in Table 2. All the synthesized compounds 5a–j showed the potential to modulate AChE activity. The four spiro compounds 5c, 5d, 5e and 5f with IC_50_ values of 4.50 ± 0.18, 7.72 ± 0.13, 5.27 ± 0.28 and 3.20 ± 0.16 μM, respectively, bearing chloro and fluoro substitution on the phenyl ring displayed excellent activity comparable to the reference drug (IC_50_ 2.09 ± 0.11), and it is observed that these four compounds showed activity with lower concentrations of even less than 10 μM. Similarly, compound 5j bearing the nitro group in the phenyl ring exhibited relatively better AChE activity with IC_50_ value of 10.40 ± 0.12 μM, while other compounds 5a–b, 5g, 5h and 5i disclosed good to moderate activities from 18.26 to 24.55 μM. Similarly, all the spiro compounds showed significant BChE inhibitory activity with IC_50_ values ranging from 18.14 ± 0.06 to 30.15 ± 0.10 μM. Compounds 5f (18.14 ± 0.06), 5d (21.02 ± 0.18) and 5j (20.16 ± 0.20) had better BChE activities comparable to those of the standard drug galantamine (19.34 ± 0.17 μM), whereas compounds 5b (24.12 ± 0.16) and 5c (22.16 ± 0.14) had good activity, and other compounds 5a (28.12 ± 0.25), 5g (29.80 ± 0.12), 5h (30.15 ± 0.10) and 5i (29.18 ± 0.12) showed comparatively less activity. The maximum BChE potency was observed for the molecule 5f (18.14 ± 0.06) bearing fluoro on the phenyl ring. The results concluded that the presence of halogenated atoms on the phenyl had a notable influence on the inhibitory activities. The results identify compound 5f as a potential candidate for further analysis using docking and MD simulations compared with the standard drug galantamine. Overall, the results of this study confirm that the compound with an electron withdrawing group exhibited significant activity.

**Table:** Details of simulated systems

S. No.	System	No. of atoms in protein	No. of atoms in the drug molecule	No. of water molecules	No. of ions		Total no. of atoms	Simulation time (ns)
					Na^+^	Cl^−^		
1	AChE	8261	—	29 068	95	87	95 647	150
2	AChE + galantamine	8261	43	29 056	95	87	95 654	150
3	AChE + 5f	8261	72	29 055	95	87	95 680	150
4	BChE	8302	—	29 069	87	91	95 687	150
5	BChE + galantamine	8302	43	29 053	87	91	95 682	150
6	BChE + 5f	8302	72	29 050	87	91	95 702	180

**Table:** AChE/BChE inhibitory activities of 5a–j

Entry	Compound	AChE inhibition IC_50_ μM (±SD)	BChE inhibition IC_50_ μM (±SD)	AChE selectivity	BChE selectivity
1	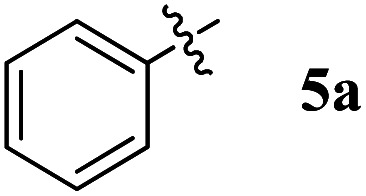	22.20 ± 0.20	28.12 ± 0.25	1.27	0.79
2	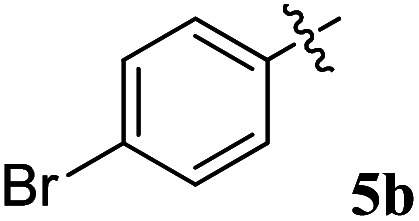	18.26 ± 0.16	24.12 ± 0.16	1.32	0.76
3	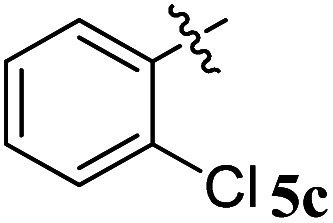	4.50 ± 0.18	22.16 ± 0.14	4.92	0.20
4	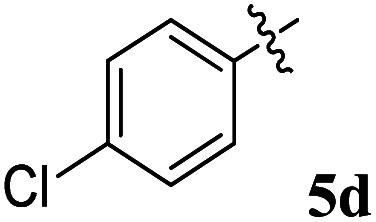	7.72 ± 0.13	21.02 ± 0.18	2.72	0.37
5	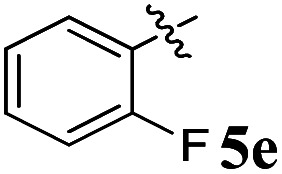	5.27 ± 0.28	20.18 ± 0.24	3.83	0.26
6	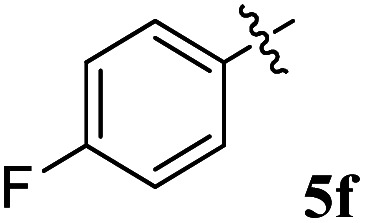	3.20 ± 0.16	18.14 ± 0.06	5.67	0.18
7	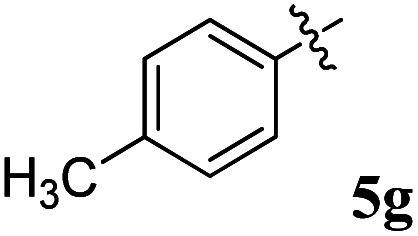	21.52 ± 0.14	29.80 ± 0.12	1.38	0.72
8	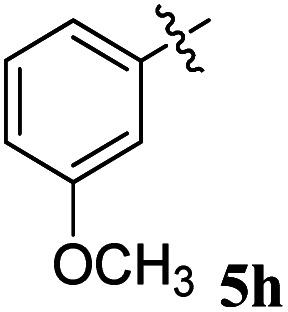	24.2 ± 0.20	30.15 ± 0.10	1.25	0.80
9	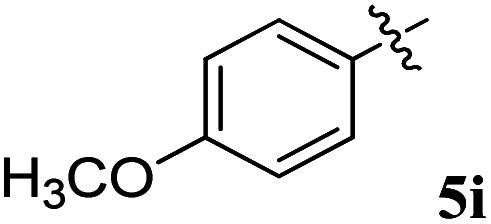	22.55 ± 0.08	29.18 ± 0.12	1.29	0.77
10	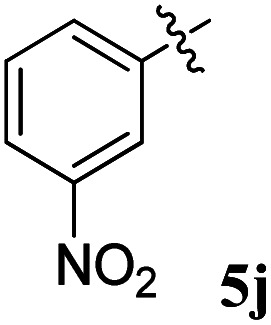	10.40 ± 0.12	20.16 ± 0.20	1.93	0.52
11	Galantamine	2.09 ± 0.11	19.34 ± 0.17	9.10	0.23

### 2.3. Docking and simulation

We performed a docking analysis to evaluate the binding efficiency of compounds 5f and galantamine with AChE and BChE. The results show that 5f has a higher binding affinity (−10.5 kcal mol^−1^) compared to galantamine (−7.56 kcal mol^−1^) with AChE. The binding affinity of 5f (−11.6 kcal mol^−1^) is higher compared to galantamine (−7.91 kcal mol^−1^) with BChE. In both cases, the best docking confirmation was identified based on the binding energy (Table 3), and this binding confirmation was further used to generate the protein–ligand complex. To validate the site-specific binding affinity data, we performed molecular dynamic simulations, as outlined in the methods.

**Table:** AutoDock binding scores

Protein targets	Binding energy (kcal mol^−1^)	
	Galantamine	5f
AChE	−7.56	−10.5
BChE	−7.91	−11.6

Further, the compound of interest 5f was assessed using Swiss ADME^37^ to evaluate its absorption, distribution, and metabolism. The results predict that 5f exhibits a low absorption due to its high molecular weight, and it does not agree with the Lipinski rule but follows the Veber rule. A summary of the findings is presented in Table S1.† Many of the drugs that are currently in use do not pass the Blood–Brain Barrier (BBB); however, there are several methods to deliver such drugs, but delivering adequate quantities might still be a challenge. Poor solubility and low bioavailability can be tackled by employing techniques such as targeted drug delivery, reformulation, self-emulsifying formulations, proliposomes, or even micelle-based products. About 40% of drugs with market approval exhibit poor solubility in water. With the advent of various insoluble drug delivery technologies, the challenge of formulating poorly water-soluble drugs can be achieved. Numerous drugs associated with poor solubility and low bioavailability have been formulated as successful drug products.^38^ Once the drug is delivered using targeted delivery methods to the Central Nervous System (CNS), it prevents the drug from causing systemic toxicity.

We also conducted an analysis using Swiss Target Prediction^39^ to predict potential targets based on molecular similarity and shape. Our findings revealed that 5f has a 33.3% likelihood of targeting family A, G protein-coupled receptors, a 26.7% likelihood of targeting enzymes, a 26.7% likelihood of targeting proteases, a 6.7% likelihood of targeting cytosolic proteins, and a 6.7% likelihood of targeting electrochemical transporters. Galantamine has a 53.3% chance of targeting family A, G protein-coupled receptors, a 13.3% chance of hydrolase and nuclear receptors and a 6.7% chance of targeting electrochemical transporters, enzymes, and membrane receptors. The off-target interactions of compound 5f are better than those of the standard drug galantamine.

### 2.4. Molecular dynamics simulation results

**Fig. 4:**
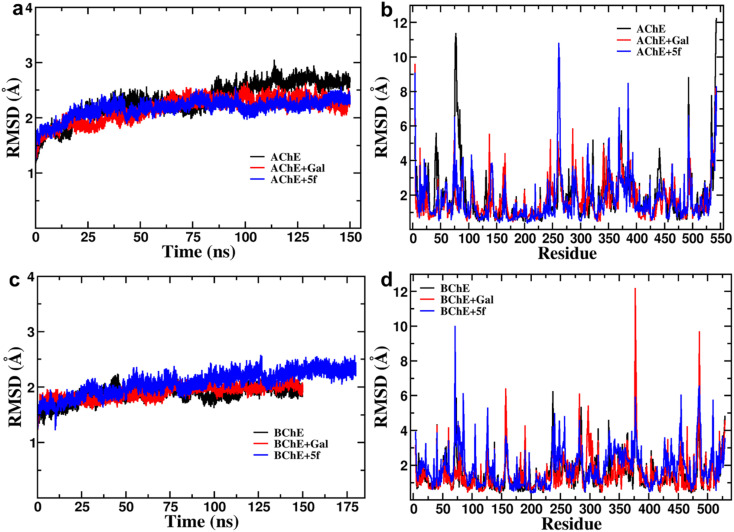
RMSD profiles of protein (AChE/BChE) as a function of (a and c) simulation time (b and d) residue numbers compared with those of proteins present in AChE/BChE + Gal and AChE/BChE + 5f complexes. As free-standing protein and protein–ligand complex structures attain a stable conformation state, all RMSD profiles become saturated after 100 ns of MD. Both drugs (galantamine and 5f) stabilize the binding site of AChE, resulting in a reduction in their RMSDs (2.23 ± 0.07 and 2.38 ± 0.05 Å) compared to the RMSD of the free-standing protein (2.65 ± 0.06 Å). In the case of BChE, galantamine stabilizes the protein binding site, whereas 5f distorts the local protein structure relative to the free-standing protein. Consequently, the RMSD of protein in the BChE + 5f (2.31 ± 0.07 Å) complex is higher compared to the RMSD of the free-standing protein (1.93 ± 0.05 Å).

**Fig. 5:**
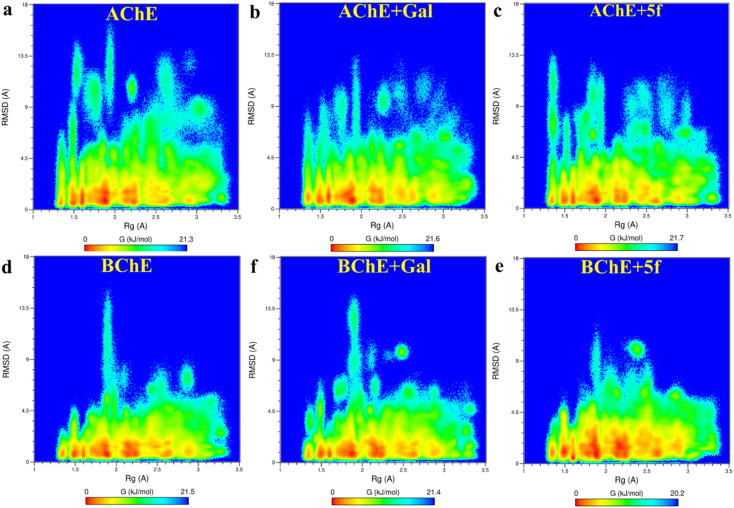
2-D free energy landscapes as a function of amino acid RMSD and *R*_g_ are obtained for proteins (AChE/BChE) in (a and d) free-standing and in those complexed with (b and f) galantamine and (d and e) 5f. The amino acids have 7 different (red) regions of Rg centered at 1.3 Å, 1.5 Å, 1.6 Å, 1.9 Å, 2.2 Å and 2.5 Å for both AChE/BChE complexes. A small reduction in intensities of the high-intensity domains of AChE + Gal and AChE + 5f relative to AChE. In contrast, the increase in the intensities of the high-intensity domains of BChE + 5f spread along RMSDs relative to that of BChE + Gal and BChE.

Root-mean-square-deviation (RMSD) profiles of free-standing proteins (AChE/BChE) are computed as a function of simulation time (Fig. 4a and c) and residue number (Fig. 4b and d). The computed values of 5f with AChE are compared with the corresponding values of galantamine with AChE. The MD results show a large conformational change observed as a change in RMSD in the protein or protein–ligand complex to adjust to the solvent environment during the first 2 ns of MD. Later, RMSD profiles gradually increased with time and became saturated after 100 ns as the protein structures reached a thermodynamic stable state. Further, the RMSDs of proteins in the AChE + Gal (2.23 ± 0.07 Å) and AChE + 5f (2.38 ± 0.05 Å) complexes are lower compared with the RMSD of free-standing protein (2.65 ± 0.06 Å). This is attributed to the stabilization of the AChE binding site by galantamine and 5f compounds, respectively, compared to free standing protein. The RMSD of protein in the BChE + 5f (2.31 ± 0.07 Å) complex is higher relative to both the RMSDs of protein in the BChE + Gal (2.04 ± 0.07 Å) complex and free-standing protein (1.93 ± 0.05 Å). These observations agree with the higher RMSD per residue observed for the region of 225–500 residues in BChE + 5f relative to BChE + Gal and free-standing protein. These results demonstrate that the binding affinity of 5f and galantamine with AChE is much stronger than that observed for BChE, which is also in agreement with the values observed in enzyme assays. Further, 2D free energy landscape (FEL) is computed as a function of RMSD and *R*_g_ per residue for free standing proteins (Fig. 5a and d) and protein–ligand complexes (Fig. 5b, c, e and f) to understand the changes in confirmations and size dependency of amino acids. Seven high-intensity regions are observed centered at 1.3 Å, 1.5 Å, 1.6 Å, 1.9 Å, 2.2 Å and 2.5 Å for all AChE/BChE complexes. There is a minute reduction in intensities of high-intensity regions of FEL for AChE + Gal (Fig. 5b) and AChE + 5f (Fig. 5c) complexes relative to that of the free-standing AChE. In contrast, the high-intensity regions of FEL become larger and thicker for the BChE + 5f (Fig. 5e) complex compared to that of BChE + Gal (Fig. 5f) and free standing BChE (Fig. 5d). This agrees with the larger RMSDs of AChE and BChE + 5f with respect to the other corresponding protein–ligand complexes.

**Fig. 6:**
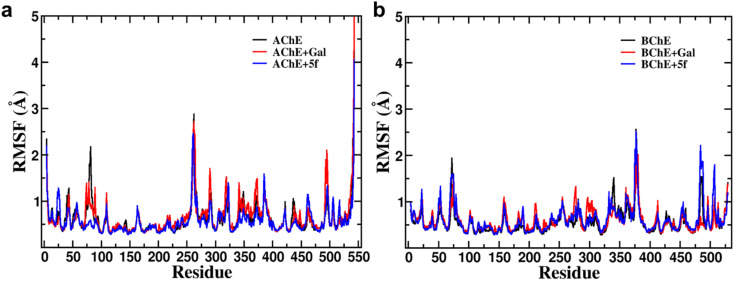
Superimposition of (a) RMSF profiles of protein (AChE) in free-standing protein, AChE + Gal and AChE + 5f complexes and (b) RMSF profiles of protein (BChE) in free-standing protein, BChE + Gal and BChE + 5f complexes. The high-intensity peaks present in the RMSF profiles of AChE/BChE reveal that the region residues 1–100, 250–400, and 480–520 are more flexible compared to the other proteins. Further, the binding of galantamine/5f with AChE reduces its flexibility, whereas its effect on the flexibility of BChE is suboptimal.

Root-mean-square-fluctuation (RMSF) as a function of residue is a good measurement for understanding protein structure flexibility. This helps to elucidate differences in changes in the fluctuations of residues as a function of ligand binding compared to free protein. The RMSF profiles are computed for compound 5f and galantamine with the enzymes AChE/BChE and compared with corresponding free-standing proteins. The results are presented in Fig. 6, respectively. The high-intensity peaks present in the RMSF profiles of AChE/BChE reveal that the regions with residues 1–100, 250–400, and 480–520 are more flexible compared to the rest of the proteins. Further, the RMSF profile of AChE is greatly suppressed by binding with the inhibitor compound 5f (see Fig. 6a) compared to the standard galantamine, while it is mildly affected for BChE (Fig. 6b). The reduction in the flexibility of AChE can be attributed to the strong binding of compound 5f relative to standard galantamine.

**Fig. 7:**
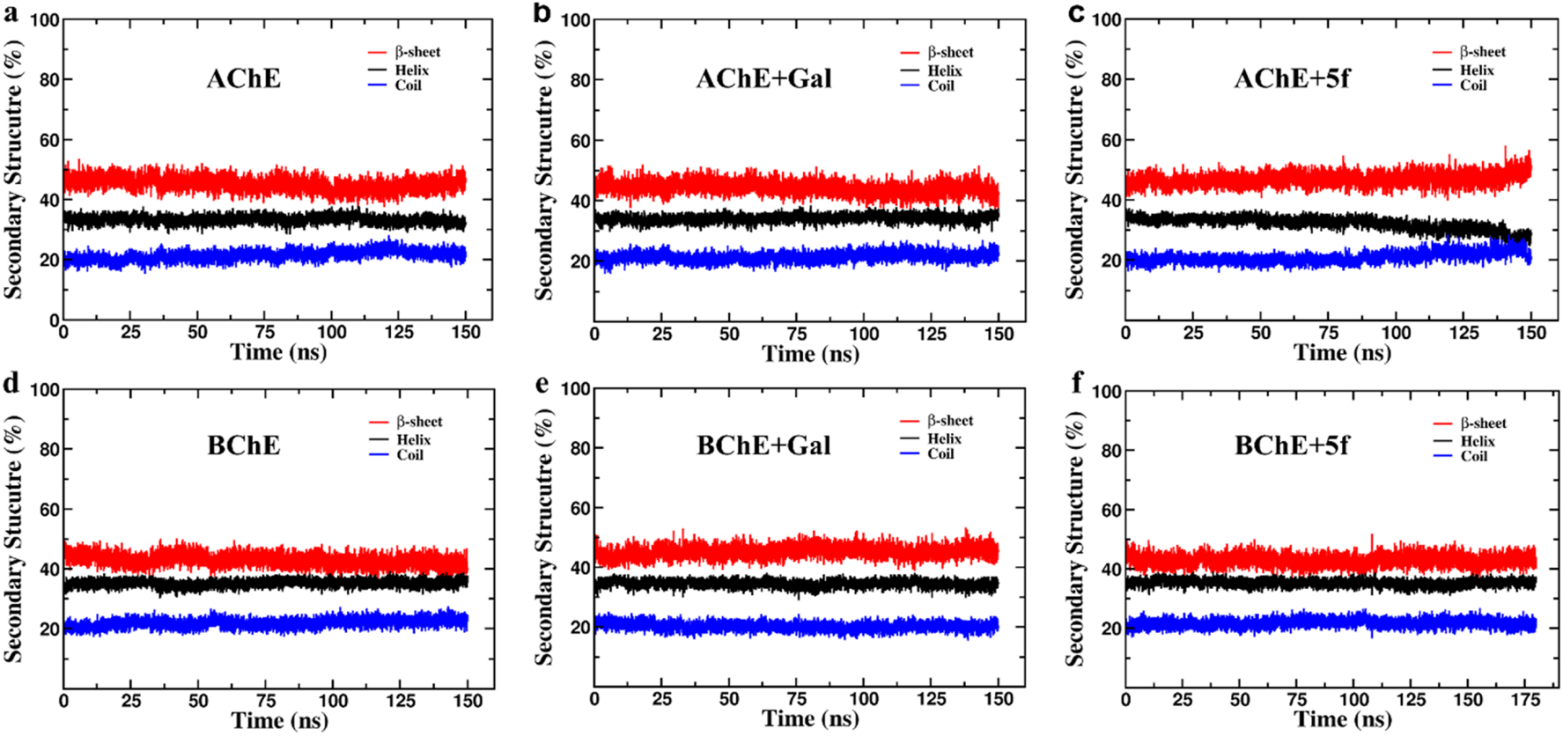
Secondary structure (SS) profiles of protein (AChE/BChE) as a function of simulation time in (a and d) free standing in complex with (b and e) galantamine and (c and f) 5f, respectively. The SS profiles of proteins in the AChE + Gal and BChE + 5f complexes are very similar to those of free-standing AChE/BChE, respectively. In contrast, the proteins in the AChE + 5f and BChE + Gal complexes undergo a 3–4% of helix/coil to β-sheet transitions with respect to their free-standing confirmations.

The secondary structure (SS) profiles of AChE/BChE are computed as a function of simulation time (Fig. 7a–f), and average values are compared (Table 4) to understand the effect of protein–ligand complexation on its structure. This concludes that the SS of AChE is composed of 33% of helix, 22% of coil and 45% of β-sheet while BChE has 35% of helix, 23% of coil and 42% of β-sheet in their free-standing protein confirmations. About 3–4% of SS of AChE/BChE undergo helix/coil to β-sheet transformations, while they form complexes with 5f/galantamine respectively (Fig. 6c and e). The SS perturbations for AChE + Gal and BChE + 5f are suboptimal and limited to 1% of their total structures. Interestingly, the protein segments of AChE (residues: 335–340, 397–407, and 532–542) and BChE (residues 276–287, 372–382, and 412–415) near their active sites are mainly responsible for these SS transitions.

**Table:** Secondary structure transitions of AChE/BChE with ligand (galantamine/5f) binding

	Helix (%)	Coil (%)	β-sheet (%)	Δhelix (%)	Δcoil (%)	Δ*β*-sheet (%)	SS transition
AChE	32.89 ± 1.18	22.11 ± 1.23	44.99 ± 1.93	—	—	—	
AChE + galantamine	34.24 ± 1.24	21.87 ± 1.32	43.89 ± 2.08	+1.35	−0.24	−1.10	β-sheet to helix
AChE + 5f	28.69 ± 1.95	22.89 ± 1.62	48.42 ± 2.37	−4.20	+0.78	+3.43	Helix/coil to β-sheet
BChE	35.46 ± 1.10	22.81 ± 1.28	41.73 ± 1.86	—	—	—	
BChE + galantamine	34.12 ± 1.12	20.11 ± 1.21	45.77 ± 1.83	−1.34	−2.70	+4.04	Helix/coil to β- sheet
BChE + 5f	35.53 ± 0.92	21.38 ± 1.25	43.09 ± 1.70	+0.07	−1.43	+1.36	Coil to β-sheet

**Fig. 8:**
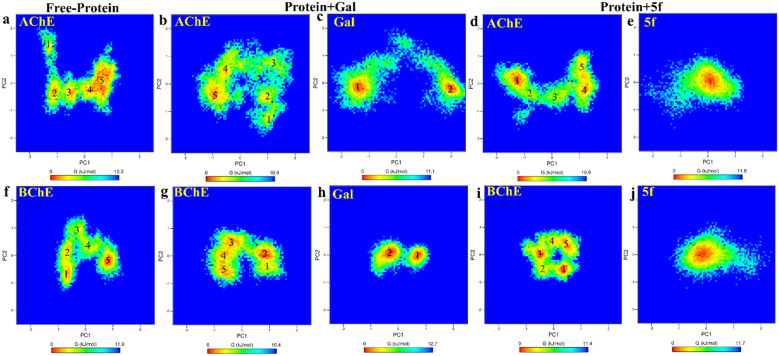
2D-free energy landscape of free-standing proteins ((a) AChE and (f) BChE), protein + galantamine ((b) AChE and (c) Gal in AChE + Gal and (g) BChE and (h) Gal in BChE + Gal) complexes and protein + 5f ((d) AChE and (e) 5f in AChE + 5f and (i) BChE and (j) 5f in BChE + 5f) complexes are obtained as a function of PC1 and PC2. The free energy minima of AChE spread longer along PC1 and PC2 relative to that of BChE in comparison with the respective complexes. The differences are maximum for protein + Gal complexes. This can be attributed to the lesser binding affinity of galantamine with AChE compared with BChE.

Further, the free energy landscapes (FEls) of AChE/BChE and their complexes with galantamine and 5f are obtained as a function of their PC1 and PC2, as shown in Fig. 8a–j, to understand the differences in various thermodynamical favorable states acquired in connection with their structure and binding. All FELs show five minima (labeled 1–5) corresponding to different thermodynamically favorable conformational states of AChE/BChE. The FELs of AChE increase more along PC1 and PC2 than the corresponding FELs of BChE, and these differences become larger in the cases of the protein–ligand complexes compared to the free-protein structures. This is due to the perturbation of protein structures by the ligand binding, which leads to various thermodynamically favorable states. Further, the FELs of galantamine pose two minima (Fig. 8c and h), while they become a single global minimum for 5f (Fig. 8e and j) because of their larger molecular size relative to galantamine. Consequently, the protein states in the FELs of AChE + Gal (Fig. 8b) and BChE + Gal (Fig. 8g) are widely distributed along PC1 compared to those of AChE + 5f (Fig. 8d) and BChE + 5f (Fig. 8i), respectively.

**Fig. 9:**
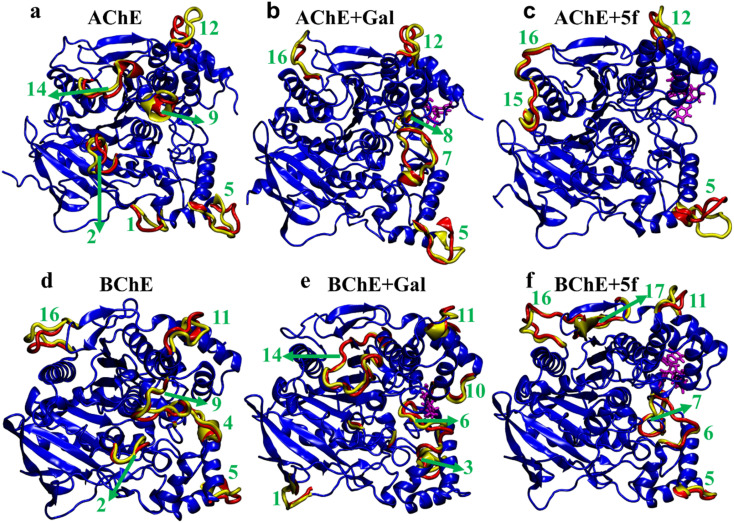
Regions that majorly contribute to the structural transitions of protein in (a and d) free-standing (AChE and BChE), (b and e) protein + galantamine (AChE + Gal and BChE + Gal), and (c and f) protein + 5f (AChE + 5f and BChE + 5f) complexes. A total of 10 and 14 regions of AChE and BChE, respectively, are involved in the structural transitions between various thermodynamically favorable states of protein–ligand complexes.

To understand transitions between the protein states that appeared in FELs and their connection with their structural changes, we identify the different protein regions (labeled according to Table 5) that are majorly involved in the AChE/BChE transitions, as shown in Fig. 9a–f. Additionally, these protein regions are tabulated in Table 6 for all different cases based on their involvement in transitions. Interestingly, a large number of (5–6) protein segments are responsible for free-standing AChE and BChE + Gal transitions, while they are limited to 2–4 protein segments in the other cases. It is observed that loop regions 5 or 11 of AChE or BChE mostly appeared in all their transitions. A smaller number of protein segments are involved in FEL BChE transitions, and the close compact distribution of minima compared to that of AChE can be explained by the relatively lower RMSDs observed for BChE complexes.

**Table:** Definition of various regions of the AChE/BChE structures

Region	1	2	3	4	5	6	7	8	9	10	11	12	13	14	15	16	17
AChE	41–46	77–87	—	—	256–266	—	287–298	317–320	340–346	—	—	383–390	—	435–443	460–466	491–496	—
BChE	50–57	72–76	156–162	235–239	251–258	276–287	295–303	—	335–344	358–362	372–382	—	412–415	423–438	—	481–497	500–504

Regions that are involved in the AChE/BChE structure transitions in various protein–ligand complexes

**Table:** 

Transition	AChE (regions)	AChE + Gal (regions)	AChE + 5f (regions)
1–2	2, 5, 9, 12, 14	5, 7, 16	5, 12
2–3	2, 5, 9, 12, 14	5, 8, 12, 16	5, 15
3–4	5, 9, 12, 14	5, 8, 16	5, 15
4–5	1, 2, 5, 12, 14	5, 16	5, 15, 16

**Table:** 

Transition	BChE (regions)	BChE + Gal (regions)	BChE + 5f (regions)
1–2	5, 9, 16	1, 10, 11, 14	10, 11, 16
2–3	2, 9, 11	2, 3, 6, 11, 14	10, 11, 16
3–4	2, 4, 6, 9	2, 6, 10, 11, 14	6, 7, 10, 11, 16, 17
4–5	2, 9, 11, 16	1, 3, 6, 11, 13, 14	5, 11, 16

**Fig. 10:**
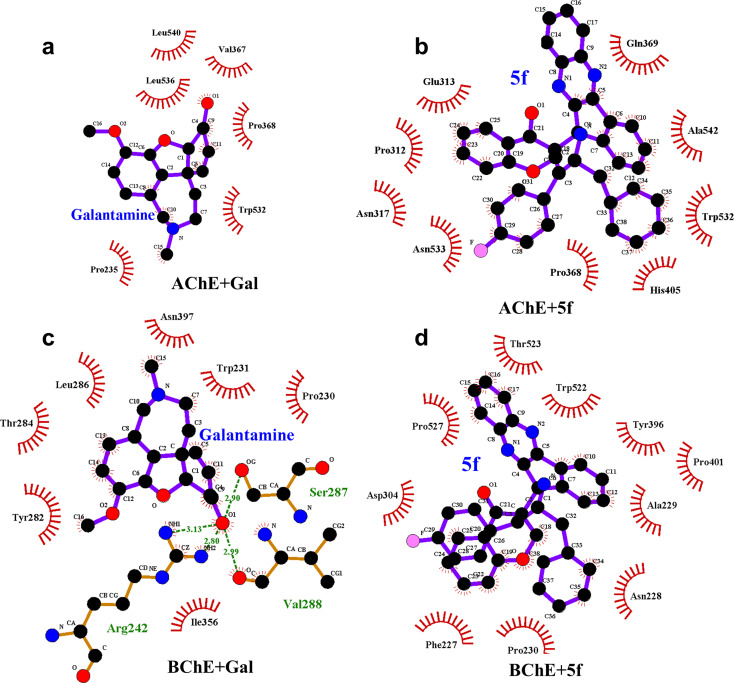
LIG plots of (a) Gal + AChE, (b) 5f + AChE, (c) Gal + BChE and (d) 5f + BChE in corresponding protein–ligand complexes. The number of non-bonding interaction residues (9) present in the LIG plot of the AChE+5f complex is higher compared to the number of nonbonding interaction residues (6) present in the LIG plot of the AChE + Gal complex. Contrarily, LIG plots of both BChE + Gal and BChE + 5f complexes have the same number of (10) nonbonding interaction residues.

The differences in structure, FELs and flexibility of proteins as a function of ligand binding can be understood by probing the residues that interact with them locally in the binding site. To understand ligand–protein interaction differences, the LIG plots are generated and compared, as shown in Fig. 10. The results reveal that a total of nine non-bonding interaction residues are observed for the AChE + 5f complex, which is much higher compared to that of 6 interacting residues in the case of the AChE + Gal complex. The strength of non-bonding interactions directly influences the binding affinity of the protein and ligand. High binding affinity is crucial for effective drug molecules because they form stable complexes that are necessary for their biological functions. In summary, non-bonding interactions show how strong the protein–ligand complexes are and how important they are for accurate docking predictions. However, 5f is a larger molecule than galantamine, and consistent with its size, a larger number of interactions are envisaged for 5f, which is reflected in the study (Fig. 10). Further, the lower number of interaction residues of AChE near galantamine than that of 5f can be attributed to the higher intensity of the RMSF peaks in the case of AChE + Gal compared to that of the AChE + 5f complex. In contrast to the AChE, the LIG plots of both BChE + 5f and BChE + Gal show a total of ten non-bonding interaction residues. This is consistent with the similarity in RMSF profiles observed with the 5f and Gal–protein complexes of BChE. Taken together, our results show considerable concordance in the results observed for docking and the MD for 5f and galantamine.

**Fig. 11:**
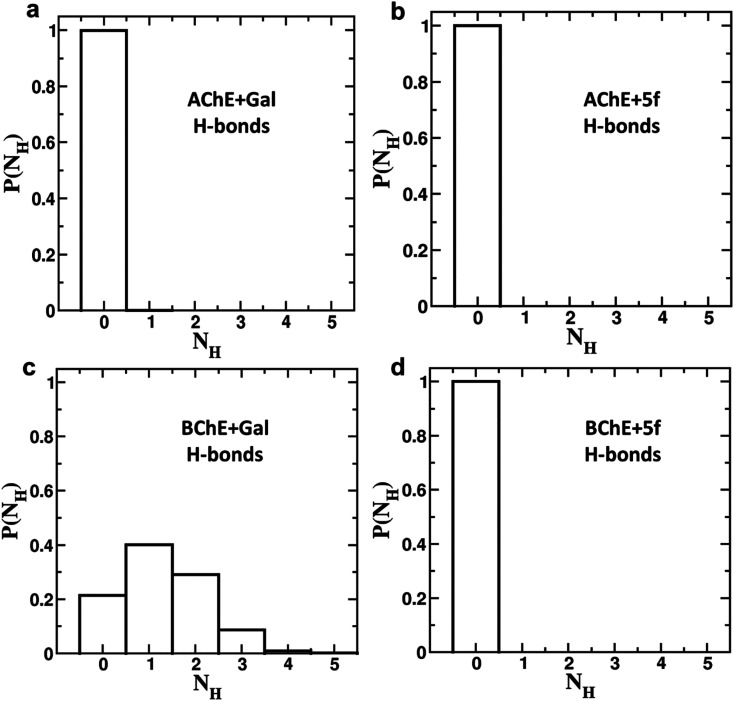
Hydrogen bond formation probabilities of (a) galantamine (Gal) and (b) 5f with AChE, as well as (c) galantamine (Gal) and (d) 5f with BChE in the corresponding protein–drug complexes. The hydrogen bond analysis confirms that single, double and tribble hydrogen bonds can be observed between galantamine–BChE with probabilities of 0.4, 0.3, and 0.1, respectively. In all other cases, no hydrogen bonds were observed between the protein and ligand molecules.

To understand the differences in the protein structure, flexibility, and local interaction environment, the MMPBSA calculations are performed to compute the binding free energies of 5f and galantamine compounds with AChE/BChE, as tabulated in Table 7. It is observed that the binding free energy of compound 5f (−93.5 ± 11.4 kcal mol^−1^) with AChE is much higher than that of standard galantamine (−66.2 ± 12.3 kcal mol^−1^). These differences can be attributed mainly to variations in their vdW/polar solvation energy contributions. This explains the lower peak intensities of the RMSF profiles and the higher number of non-bonding interaction residues in the LIG plot that corresponds to AChE + 5f relative to AChE + Gal. In contrast, the binding free energies of both 5f (−98.1 ± 11.2 kcal mol^−1^) and galantamine (−97.9 ± 11.5 kcal mol^−1^) compounds with BChE are similar. This can be attributed to the similarity in the local interaction environment present at the 5f/galantamine–BChE interface. Further, the amino acids contributing to the negative/positive (represented in yellow/red, respectively) binding free energies of protein–ligand complexes are shown in Fig. S6 (*vide* ESI†). The corresponding binding free energy profiles as a function of residue number with the 5f/galantamine–BChE are compared, as shown in Fig. S7 (*vide* ESI†). These results reiterate that the variations in the observed total binding free energies of the protein–ligand complexes in the case of 5f and galantamine can be attributed to differences in the number of interacting residues and their binding strengths. To probe these aspects further, we generated Venn diagrams with amino acid residues of AChE/BChE that interact with 5f/galantamine in the protein–ligand complex, as shown in Fig. S8 (*vide* ESI†). The Venn diagrams confirm that the number of residues of AChE favoring binding with 5f and galantamine are 14 and 10, respectively, while they become 12 and 13 for the BChE. These differences in the number of residues explain the observed differences in the number of close contacts and vdW energy contributions. Although the binding energies of Gal and 5f with BChE are the same, the numbers of non-bonding hydrophobic interactions are 3 and 7, respectively. However, an analysis of the hydrogen bond interaction shows that (Fig. 11) galantamine can form 1–3 hydrogen bonds with BChE with a probability in the range of 10–40%. The extra 4th hydrogen has a very low probability. These results could explain the similarity in the binding of galantamine and 5f to BChE. Consequently, our computational analysis shows differences in the interaction of 5f and galantamine with AChE and BChE. These results have consequences for the observed inhibition of AChE and BChE with implications for disease. We found considerable concordance between the results of docking and MD simulations. However, differences in activity between the IC_50_ values of galantamine and compound 5f are very similar; these differences may be attributed to the pH maintained at pH 8. The docking was performed after adding a charge to both the protein and ligand. This process is much different from what is observed in the solution. The MD simulation was performed at pH 7.2. The MD simulations provide a binding affinity that is reflective of the solvated state and active state of the protein at a given pH. We believe that the experimental conditions used might also influence the outcome of binding. Hence, the docking and MD simulation results should be considered a reflection of trends that depicts 5f binding either as equal to or higher than that of galantamine.

**Table:** Binding energies of the protein–drug complexes

Binding energy	Protein–drug complex			
	AChE + galantamine (kJ mol^−1^)	AChE + 5f (kJ mol^−1^)	BChE + galantamine (kJ mol^−1^)	BChE + 5f (kJ mol^−1^)
vdW	−117.8 ± 9.1	−196.2 ± 11.4	−156.3 ± 10.9	−185.4 ± 10.1
Electrostatic	−6.2 ± 6.3	−21.9 ± 5.5	−47.1 ± 8.7	−10.1 ± 5.6
Polar solvation	70.7 ± 13.0	146.5 ± 13.0	120.9 ± 9.9	114.0 ± 12.7
SASA	−13.0 ± 0.9	−22.5 ± 1.1	−15.4 ± 0.7	−16.6 ± 1.0
Total	−66.2 ± 12.3	−93.5 ± 11.9	−97.9 ± 11.5	−98.1 ± 11.2
No. of close contacts	648.0 ± 64.1	981.0 ± 66.4	970.0 ± 70.6	837.0 ± 56.0

## 3. Conclusion

Several dispiroindnopyrrolidine engrafted chromanones are achieved in good yields using a multicomponent cycloaddition strategy. A spectroscopic and single-crystal XRD study provided undeniable evidence of stereo and regiochemistry assignments for compounds. The AChE/BChE study revealed that four compounds were identified as having significant inhibitory activity. It is interesting to note that the compound with a fluorine atom on the aryl ring showed the highest potency. These results are comparable to those of the standard drug galantamine.

Our analysis shows that compound 5f displays good inhibitory activity against AChE, which is comparable to galantamine (3.2 *vs.* 2.09 μM). Although the inhibitory effects of compound 5f and galantamine on AChE were similar, docking studies demonstrated that the binding energy of compound 5f was −10.5 compared to galantamine 7.56 for AChE. Interestingly, the MD simulation MMPBSA results showed −93 kcal mol^−1^ for compound 5f compared to −66 kcal mol^−1^ for galantamine, which agrees more with the docking studies. It is interesting to note that while galantamine 6 residues interact with galantamine, a total of 9 residues interact with compound 5f in AChE. In the case of galantamine, a total of 7 residues interact hydrophobically compared to 10 residues in compound 5f with BChE. Interestingly, 4 hydrogen bonds are formed by galantamine with BChE but not with AChE. Further, the larger spread of the free energy minima distribution along PC1 and PC2 in the FELs of AChE + Gal relative to that of AChE + 5f, BChE + Gal and BChE + 5f complexes. This explains the differences in the binding energy of galantamine, which is comparable to 5f in the case of BChE but not with AChE. In addition, the RMSF values of galantamine/5f showed reduced flexibility in AChE compared to BChE, which explains the better inhibitory effect on AChE compared to BChE. Although there are shortcomings associated with the 5f in ADME studies, technological advancements enable targeted drug delivery of compounds to CNS.

Therefore, the enzyme inhibitor activity observed in enzyme assays and binding activity observed from docking and MD studies explain the potential of compound 5f towards AChE and BChE.

## 4. Materials and methods

An equimolar mixture of ninhydrin 1, *o*-phenylenediamine 2, and l-phenylalanine 3, followed by 3-(4-benzylidene) chroman-4-oned, 5a–j (1 mmol) in [Bmim]Br (3 ml), was heated to 100 °C for one hour. To ensure the absence of starting materials, EtOAc was extracted from the reaction mixture and concentrated. In the final step, the cycloadduct is washed with Et_2_O and filtered to obtain a product in pure form. Recrystallization from ethyl acetate provided the single crystals.

### 4.1. 5-Benzyl-4-(2-chlorophenyl)-2-spiro[2,6′′]indenoquinoxalino-3-spiro[3,3′]-chromanonopyrrolidine, 5c

^1^H NMR: *δ*_H_ 3.11 (d, *J* = 6.5 Hz, 2H), 3.35 (d, *J* = 13.5 Hz, 1H), 4.01 (d, *J* = 12.0 Hz, 1H), 5.14 (d, *J* = 9.0 Hz, 1H), 5.31–5.35 (m, 1H), 6.21 (d, *J* = 8.0 Hz, 1H, ArH), 6.76 (t, *J* = 7.5 Hz, 1H, ArH), 7.02–7.05 (m, 1H, ArH), 7.07–7.10 (m, 2H, ArH), 7.14–7.19 (m, 4H, ArH), 7.23–7.30 (m, 4H, ArH), 7.36–7.40 (m, 1H, ArH), 7.74–7.78 (m, 2H, ArH), 7.81–7.82 (m, 1H, ArH), 7.85 (d, *J* = 7.5 Hz, 1H),8.11–8.13 (m, 1H, ArH), 8.22–8.24 (m, 1H, ArH), 8.61 (d, *J* = 8.0 Hz, 1H, ArH); ^13^C NMR: *δ*_C_ 40.7, 49.4, 61.0, 64.7, 72.2, 73.7, 116.9, 121.1, 121.2, 121.8, 126.3, 126.7, 128.2, 128.4, 129.2, 129.3, 129.4, 129.6, 129.9, 135.1, 135.2, 136.3, 136.9, 138.7, 140.4, 142.2, 145.3, 154.0, 160.6, 163.7, 192.7; mass *m*/*z*: 605 (M^+^); Anal. Calcd for C_39_H_28_ClN_3_O_2_: C, 77.28; H, 4.66; N, 6.93; found C, 77.39; H, 4.78; N, 7.04%.

### 4.2. 5-Benzyl-4-(2-fluorophenyl)-2-spiro[2,6′′]indenoquinoxalino-3-spiro[3,3′]-chromanonopyrrolidine 5d

^1^H NMR: *δ*_H_ 3.05–3.25 (m, 2H), 3.38–3.46 (m, 1H), 4.25 (d, *J* = 10.0 Hz, 1H), 5.05–5.07 (m, 1H), 5.30–5.33 (m, 1H), 6.18–20 (m, 1H, ArH), 6.70–6.72 (m, 1H, ArH), 7.00–7.04 (m, 2H, ArH), 7.11–7.27 (m, 12H, ArH), 7.74–7.80 (m, 4H, ArH), 8.11–8.12 (m, 1H, ArH), 8.21–8.24 (m, 1H, ArH), 8.30–8.32 (m, 1H, ArH); ^13^C NMR: *δ*_C_ 40.4, 44.1, 60.8, 63.1, 72.1, 73.0, 115.5, 121.1, 121.8, 123.8, 124.2, 126.4, 126.7, 128.1, 128.4, 128.8, 129.2, 129.4, 129.7, 130.5, 130.9, 135.1, 136.9, 138.7, 140.5, 142.2, 145.7, 153.9, 160.8, 164.2, 192.4; mass *m*/*z*: 589 (M^+^); Anal. Calcd for C_39_H_28_FN_3_O_2_: C, 79.44; H, 4.79; N, 7.13; found C, 79.56; H, 4.90; N, 7.25%.

### 4.3. 5-Benzyl-4-(4-fluorophenyl)-2-spiro[2,6′′]indenoquinoxalino-3-spiro[3,3′]-chromanonopyrrolidine, 5e

^1^H NMR: *δ*_H_ 2.90–2.94 (dd, *J* = 14.5, 8.0 Hz, 1H, ArH), 3.12–3.15 (dd, *J* = 14.5, 4.0 Hz, 1H), 3.29 (d, *J* = 12.0 Hz, 1H), 4.62 (d, *J* = 12.5 Hz, 1H), 4.68 (d, *J* = 10.0 Hz, 1H), 5.16–5.21 (m, 1H), 6.15 (d, *J* = 8.5 Hz, 1H, ArH), 6.65 (d, *J* = 9.5 Hz, 1H), 7.00–7.24 (m, 13H, ArH), 7.47–7.49 (m, 1H, ArH), 7.71–7.77 (m, 3H, ArH), 8.09–8.11 (m, 1H, ArH), 8.21–8.23 (m, 1H, ArH); ^13^C NMR: *δ*_C_ 39.8, 51.2, 61.4, 62.6, 71.8, 71.9, 115.6, 115.7, 116.7, 129.9, 121.6, 126.4, 126.6, 127.7, 128.5, 128.9, 129.2, 129.3, 129.4, 129.6, 129.7, 130.0, 132.1, 135.3, 136.7, 138.5, 140.7, 142.2, 146.7, 153.7, 160.2, 161.2, 163.2, 165.0, 192.7; mass *m*/*z*: 589 (M^+^); Anal. Calcd for C_39_H_28_FN_3_O_2_: C, 79.44; H, 4.79; N, 7.13; found C, 79.54; H, 4.87; N, 7.22%.

### 4.4. 5-Benzyl-4-(4-chlorophenyl)-2-spiro[2,6′′]indenoquinoxalino-3-spiro[3,3′]-chromanonopyrrolidine, 5f

^1^H NMR: *δ*_C_2.90–2.96 (dd, *J* = 14.0, 8.0 Hz, 1H, ArH), 3.11–3.15 (dd, *J* = 14.0, 4.0 Hz, 1H), 3.31 (d, *J* = 12.8 Hz, 1H), 4.61 (d, *J* = 12.8 Hz, 1H), 4.67 (d, *J* = 11.2 Hz, 1H), 5.17–5.22 (m, 1H), 6.17 (d, *J* = 8.0 Hz, 1H, ArH), 6.66 (d, *J* = 7.6 Hz, 1H), 7.00–7.05 (m, 1H), 7.13–7.26 (m, 10H, ArH), 7.35–7.37 (m, 2H, ArH), 7.48–7.51 (m, 1H, ArH), 7.73–7.79 (m, 3H, ArH), 8.10–8.13 (m, 1H), 8.22–8.24 (m, 1H, ArH); ^13^C NMR: *δ*_C_ 39.7, 51.1, 61.3, 62.5, 71.7, 71.8, 115.6, 116.6, 120.8, 126.3, 128.4, 128.8, 129.1, 129.2, 129.3, 129.5, 129.5, 129.6, 130.9, 135.2, 136.6, 138.4, 140.6, 142.1, 146.6, 153.6, 160.1, 161.1, 163.0, 164.9, 192.6; mass *m*/*z*: 605 (M^+^); Anal. Calcd for C_39_H_28_ClN_3_O_2_: C, 77.28; H, 4.66; N, 6.93; found C, 77.37; H, 4.79; N, 7.02%.

### 4.5. Molecular docking and molecular dynamics simulation methodology

In this work, we used acetyl and butyrylcholinesterases as the target proteins to determine the effect of compound 5f as an inhibitor in comparison with galantamine, a known cholinesterase inhibitor. AChE exists in multiple forms, including monomers, dimers, and tetramers.^40–43^ Using an *in silico* molecular docking study, we evaluated the binding capacity of compound 5f when docked with human acetylcholinesterase (hAChE) and butyrylcholinesterase (hBChE) as targets. The ligand of interest 5f was converted to 3D coordinates using an open-source online tool called OpenBabel (OPENBABEL – Chemical file format conversion (https://www.cheminfo.org)). The ligand was energy minimized using Schrödinger Release-Maestro Schrodinger. The 3D coordinates of the target proteins were taken from RCSB PDB (https://www.rcsb.org/). PDB id: 7RB5 (hAChE) and 6QAA (hBChE) were cleaned of water and heteroatoms using BIOVIA, Dassault Systèmes; energy minimization was performed using the FoldX repairPDB.^44,45^ The active sites of the target proteins were identified using CASTp^46^ (http://sts.bioe.uic.edu/castp/).

A common cholinesterase inhibitor, galantamine (PubChem CID 9651), was used as a standard compound for docking and comparing the binding energy with the compound of interest. AutoDock and Cygwin ^47^ were used to conduct the docking study. AutoDock^47^ was used to add hydrogen atoms and Kollman charges to the protein molecules. Both the protein and ligand are converted to the pdbqt docking format. Docking runs were set for 10 numbers, and the protein–ligand confirmation corresponding to the best docking score was used for the MD simulation studies.

The docked protein–ligand complexes were subjected to 150 ns of production MD to obtain the thermodynamically stable structures. The system building and simulation protocol employed in MD are expounded herein.

The docked protein–ligand complexes were solvated using SPC/E,^48^ and a 1.0 nm thick water layer was maintained around the solute in all three directions (*X*, *Y* and *Z*). The solvated complexes were neutralized by adding the appropriate number of counter ions, in addition to which Na^+^ and Cl^−^ ions were added to maintain a concentration of 0.15 M close to the physiological salt concentration. The simulated system details are presented in Table 1. All-atom MD simulations were performed using the GROMACS^49^ software package. The AMBER compatible general amber force field^50^ parameters were parameterized using an antechamber and used for the ligand (5f). Amber99SB-ILDN^51^ force field was employed for the protein. The protein–ligand complex systems were subjected to the 50 000 steps of steepest descent minimization to remove the bad contacts between the protein, ions, and water atoms. The energy-minimized structures were equilibrated for 100 ps *NVT* (at constant number–volume–temperature) ensemble MD, followed by a 100 ps *NPT* (at constant number–pressure–temperature) ensemble MD under ambient conditions with a temperature of 300 K and a pressure of 1.0 atm. The constant temperature and pressure of the systems were achieved using the Berendsen^52^ thermostat and Parrinello-Rahman^53^ barostat, respectively. The protein atoms were restrained to their initial positions with a force constant of 1000 kcal mol^−1^ Å^−2^ to reach thermodynamic equilibrium with the solvent environment. Later, the equilibrated systems were subjected to a 150 ns production MD (BChE + 5f was run for 180 ns to reach equilibration) by removing the position restraints of protein atoms. The bonds involving H-atoms are constrained with harmonic restraints using LINCS^54^ algorithm to achieve the 2 fs time step of MD. The short-range non-bonding (van der Waals (vdW) and Coulomb) interactions are computed with a cut-off of 1.2 nm, and long-range electrostatic interactions were computed using the Particle-Mesh-Ewald (PME)^55^ method. The above-mentioned MD protocol was used to obtain the stable MD trajectories of various systems^56–62^ involving proteins and lipid membranes. Various structural properties, such as final snapshots, RMSDs, RMSF, RMSD per residue, number of hydrogen bonds, free-energy landscape (FELs), protein-ligand interactions, and binding energies, were obtained by analyzing production MD trajectories.^63^ SS was calculated by implementing the structural codes provided by the STRIDE^64^ algorithm in Visual Molecular Dynamics (VMD) software.^65^ The last 10 ns trajectory was used to compute equilibrium properties, such as RMSD per residue, RMSF, and number of H-bonds. Molecular Mechanics/Poisson–Boltzmann Surface Area (MMPBSA)^66,67^ was employed to compute various contributions (vdW, solvation, and surface accessible surface area (SASA)) of protein-ligand binding energies. Protein–ligand LIG plots were generated using LigPlot+^68^ with the final MD structures. PCA-based 2D Free Energy Landscapes (FELs) were generated for protein (AChE/BChE) and ligands (5f/GAL) separately using the covar, anaeig, and sham modules in GROMACS. Initially, Cα atoms of protein were used to align the structure, and a covariance matrix was formed using the covar module to obtain the eigenvalues and eigenvectors. Then, an anaeig module was used to project the trajectory on eigenvectors. In the case of FELs as a function of *R*_g_ and RMSD, the *R*_g_ and RMSD of each residue were computed and clustered for the last 20 ns. Finally, the sham module was used to generate FELs by Bolzmann inverting multi-dimensional histograms.

## Conflicts of interest

There are no conflicts to declare.

## Supplementary Material

RA-014-D4RA02432J-s001

RA-014-D4RA02432J-s002
