# Supplementary material for: Synthesis, computational docking and molecular dynamics studies of a new class of spiroquinoxalinopyrrolidine embedded chromanone hybrids as potent anti-cholinesterase agents
Source: RSC Adv. 2024 Jun 12;14(26):18815–31. doi: 10.1039/d4ra02432j (PMC11167517; doi:10.1039/d4ra02432j)
Supplement: RA-014-D4RA02432J-s001 [file RA-014-D4RA02432J-s001.pdf]

Electronic Supplementary Material (ESI) for RSC Advances.
This journal is © The Royal Society of Chemistry 2024

Synthesis, computational docking and molecular dynamics studies of new class of spiroquinoxalinopyrrolidine embedded chromanone hybrids as potent anti-cholinesterase agents

Natarajan Arumugam[a,*], Datta Darshan V M,[b] Vishal Venketesh,[b] Sai Sanwid Pradhan,[b] Anuj Garg,[c] Venketesh Sivaramakrishnan[b], Subbarao Kanchi,[c] Sakkarapalayam M. Mahalingam[d]*

[a]Department of Chemistry, College of Science, King Saud University, P.O. Box 2455, Riyadh 11451, Saudi Arabia.

[b]Disease Biology Lab, Department of Biosciences, Sri Sathya Sai Institute of Higher Learning, Prasanthi Nilayam, Andhra Pradesh, India. 515134.

[c]Department of Physics, Sri Sathya Sai Institute of Higher Learning, Prasanthi Nilayam, Andhra Pradesh, India. 515134

[d]Department of Chemistry, 720 Clinic Drive, Purdue University, West Lafayette, Indiana 47907, USA

*Corresponding Author**:** anatarajan@ksu.edu.sa (Natarajan Arumugam); mahalins@purdue.edu (Sakkarapalayam M. Mahalingam)

5-Benzyl-4- (4-methoxyphenyl)-2-spiro[2.6'']indenoquinoxalino-3-spiro[3.3']-chromanonopyrrolidine, ***5i***

Obtained as white solid (87%)^{1}H NMR ($CDCl_3$, 500 MHz): δ_H 2.87-2.91 (dd, *J* =14.00, 8.00 Hz), 3.14-3.17 (dd, *J* = 14.0, 3.0 Hz), 3.31 (d, *J* = 12.0 Hz, 1H), 3.78 (s, 3H), 4.62-4.66 (m, 1H), 5.15-5.19 (m, 1H), 6.12 (d, *J* = 8.0 Hz, ArH, 1H), 6.90-6.92 (m, ArH, 2H), 6.97-7.00 (m, ArH, 1H), 7.10-7.25 (m, ArH, 9H), 7.44-7.46 (m, ArH, 1H), 7.68-7.73 (m, ArH, 3H), 8.07-8.08 (m, ArH, 1H), 8.21-8.23 (m, ArH, 1H); ^{13}C NMR ($CDCl_3$, 125 MHz): δ_C 39.7, 51.2, 55.3, 61.5, 62.4, 71.9, 114.2, 116.6, 120.8, 121.0, 121.5, 126.4, 127.7, 128.5, 129.2, 129.3, 129.4, 129.6, 131.0, 135.1, 136.7, 138.8, 140.7, 142.2, 147.0, 153.8, 159.0, 160.2, 165.2, 192.8. Mass: *m/z* 601 (M^+). Anal. Calcd. For $C_{40}H_{31}N_3O_3$: C, 79.85; H, 5.19; N, 6.98 %; Found: C, 79.97; H, 5.30; N, 7.05%.

5-Benzyl-4- (phenyl)-2-spiro[2.6'']indenoquinoxalino-3-spiro[3.3']-chromanonopyrrolidine, ***5a***

Obtained as white solid (90%): ^{1}H NMR ($CDCl_3$, 500 MHz): δ/ppm 2.89-2.94 (dd, *J* = 13.5, 8.5 Hz, 1H), 3.15-3.19 (dd, *J* = 13.5, 2.5 Hz, 1H), 3.30 (d, *J* = 12.0 Hz, 1H), 4.66-4.71 (m, 2H),

5.24-5.28 (m, 1H), 6.15 (d, *J* = 10.5 Hz, 1H), 6.63 (t, *J* = 7.5 Hz, 1H), 7.01 (t, *J* = 7.0 Hz, 1H), 7.12-7.29 (m, 10H, ArH), 7.37-7.39 (m, 2H, ArH), 7.47 (d, *J* = 8.0 Hz, 1H, ArH), 7.71-7.76 (m, 4H, ArH), 8.09-8.11 (m, 1H, ArH), 8.23-8.25 (m, 1H, ArH); ^{13}C NMR ($CDCl_3$, 125 MHz): δ/ppm 39.7, 51.9, 61.7, 62.2, 71.8, 71.9, 116.6, 120.8, 120.9, 121.5, 126.4, 126.6, 127.5, 127.7, 128.5, 128.8, 129.2, 129.3, 129.4, 129.6, 129.9, 131.1, 135.2, 136.4, 136.8, 138.7, 140.8, 142.2, 146.9, 153.8, 160.2, 165.1, 192.7. Mass *m/z*: 571 (M^+); Anal. Calcd for $C_{39}H_{29}N_3O_2$: C, 81.94; H, 5.11; N, 7.35; Found C, 82.02; H, 5.23; N, 7.47%.

5-Benzyl-4-(4-bromophenyl)-2-spiro[2.6'']indenoquinoxalino-3-spiro[3.3']-chromanonopyrrolidine, ***5b***

Obtained as white solid (88%): ^{1}H NMR ($CDCl_3$, 500 MHz): δ/ppm 2.90-2.95 (dd, *J* = 14.5, 9.0 Hz, 1H), 3.11-3.14 (dd, *J* = 13.5, 3.0 Hz, 1H), 3.30 (d, *J* = 12.5 Hz, 1H), 4.60 (d, J = 12.0 Hz, 1H), 4.66 (d, *J* = 11.0 Hz, 1H), 5.17-5.21 (m, 1H), 6.15 (d, *J* = 8.0 Hz, 1H), 6.65 (t, *J* = 7.5 Hz, 1H), 7.00-7.04 (m, 1H), 7.12-7.25 (m, 10H, ArH), 7.48-7.51 (m, 3H, ArH), 7.71-7.76 (m, 3H, ArH), 8.09-8.11 (m, 1H, ArH), 8.21-8.23 (m, 1H, ArH); ^{13}C NMR ($CDCl_3$, 125 MHz): δ/ppm 39.9, 51.5, 61.4, 61.5, 71.8, 71.9, 116.7, 120.9, 121.0, 121.5, 121.6, 126.5, 126.6, 127.8, 128.5, 128.9, 129.2, 129.3, 129.4, 129.7, 129.8, 131.0, 131.9, 135.3, 135.6, 136.8, 138.5, 140.6, 142.3, 146.7, 153.8, 160.2, 164.9, 192.6; Mass *m/z*: 649 (M^+); Anal. Calcd for $C_{39}H_{28}BrN_3O_2$: C, 72.00; H, 4.34; N, 6.46; Found C, 72.11; H, 4.46; N, 6.54%.

*5-Benzyl-4-(4-methylphenyl)-2-spiro[2.6'']indenoquinoxalino-3-spiro[3.3']-chromanonopyrrolidine,**5g***

Obtained as white solid (89%): ^{1}H NMR ($CDCl_3$, 500 MHz): δ/ppm 2.35 (s, 3H), 2.89-2.94 (dd, *J* = 13.5, 8.0 Hz, 1H, ArH), 3.18-3.21 (m, 1H), 3.33 (d, *J* = 12.5 Hz, 1H), 4.67(d, *J* = 4.0 Hz, Hz, 1H), 4.70 (d, *J* = 6.0 Hz, 1H), 5.22-5.26 (m, 1H), 6.14 (d, *J* = 8.5 Hz, 1H), 6.34 (t, *J* = 7.5 Hz, 1H, ArH), 7.00 (t, *J* = 9.0 Hz, 1H, ArH), 7.12-7.28 (m, 12H, ArH), 7.48 (d, *J* = 7.5 Hz, 1H), 7.71-7.76 (m, 3H), 8.09-8.11 (m, 1H), 8.24-8.26 (m, 1H); ^{13}C NMR ($CDCl_3$, 125 MHz): δ/ppm 21.2, 39.7, 51.6, 61.6, 62.2, 71.9, 72.0, 116.6, 120.8, 121.0, 121.5, 126.4, 126.7, 127.7, 128.5, 128.8, 129.2, 129.3, 129.4, 129.6, 129.9, 131.0, 133.3, 135.1, 136.8, 137.1, 138.9, 140.8, 142.2, 147.0, 153.8, 160.2, 165.2, 192.7; Mass *m/z*: 605 (M^+); Anal. Calcd for $C_{40}H_{31}N_3O_2$: C, 82.03; H, 5.33; N, 7.17; Found C, 82.12; H, 5.45; N, 7.29%.

5-Benzyl-4-(3-methoxyphenyl)-2-spiro[2.6'']indenoquinoxalino-3-spiro[3.3']-chromanonopyrrolidine, ***5h***

Obtained as white solid (85%): ^{1}H NMR ($CDCl_3$, 500 MHz): δ/ppm 2.90-2.95 (dd, *J* = 14.0, 8.0 Hz, 1H, ArH), 3.18-3.21 (dd, *J* = 13.5, 2.5 Hz, 1H), 3.35 (d, *J* = 12.0 Hz, 1H), 3.86 (s, 3H), 4.67-4.72 (m, 1H), 5.22-5.27 (m, 1H), 6.15 (d, *J* = 8.0 Hz, 1H, ArH), 6.64 (t, *J* = 8.5 Hz, 1H), 6.82-6.83 (m, 1H), 6.99-7.03 (m, 1H, ArH), 7.12-7.30 (m, 11H, ArH), 4.48 (d, *J* = 7.0 H, 1H, ArH), 7.71-7.76 (m, 3H, ArH), 8.08-8.10 (m, 1H), 8.22-8.25 (m, 1H, ArH); ^{13}C NMR ($CDCl_3$, 125 MHz): δ/ppm 39.7, 51.9, 55.4, 61.7, 62.3, 71.8, 71.9, 116.7, 120.8, 120.9, 121.6, 126.4, 126.6, 127.8, 128.5, 128.9, 129.1, 129.3, 129.4, 129.6, 129.7, 129.8, 131.0, 135.2, 136.8, 138.1, 138.7, 140.7, 142.2, 146.9, 153.8, 159.9, 160.2, 165.1, 192.7; Mass *m/z*: 605 (M^+); Anal. Calcd for $C_{40}H_{31}N_3O_3$: C, 79.85; H, 5.19; N, 6.98; Found C, 79.96; H, 5.31; N, 7.08;%.

5-Benzyl-4-(3-nitrophenyl)-2-spiro[2.6'']indenoquinoxalino-3-spiro[3.3']-chromanonopyrrolidine, ***5j***

Obtained as white solid (90%); ^{1}H NMR ($CDCl_3$, 500 MHz): δ_H 3.05-3.06 (m, 2H), 3.23 (d, *J* = 12.5 Hz), 4.42 (d, *J* = 12.5 Hz, 1H), 4.81 (d, *J* = 10.0 Hz, 1H), 5.25-5.29 (m, 1H), 6.16 (d, *J* = 8.0 Hz, 1H), 6.71 (t, *J* = 7.0 Hz, 1H, ArH), 7.00-7.24 (m, 11H, ArH), 7.62-7.64 (m, 1H, ArH), 7.74-7.82 (m, 4H, ArH), 8.07-8.11 (m, 3H, ArH); ^{13}C NMR ($CDCl_3$, 125 MHz): δ_C 40.6, 52.0, 61.1, 64.1, 67.2, 72.1, 72.5, 116.8, 120.9, 121.2, 121.8, 122.3, 122.4, 123.9, 124.2, 124.8, 126.5, 126.6, 127.9, 128.1, 128.5, 129.1, 129.3, 129.4, 129.5, 129.7, 129.9, 135.5, 136.8, 138.2, 139.4, 140.5, 142.2, 145.8, 148.6, 153.7, 160.3, 164.0, 192.7; Mass: *m/z* 601 (M^+). Anal. Calcd. For $C_{39}H_{28}N_4O_4$:C, 75.96; H, 4.58; N, 9.09%; Found: C, 76.04; H, 4.71; N, 9.20%.

In vitro cholinesterase enzymes inhibitory activity

The test samples for cholinesterase enzymes inhibitory potential was evaluated using modified Ellman's method as described by Ahmed and Gilani [1-3]. Galantamine was used as positive control. Solutions of test samples and galantamine were prepared in DMSO at an initial concentration of 1 mg/mL (1000 ppm). The concentration of DMSO in final reaction mixture was 1%. At this concentration, DMSO has no inhibitory effect on both acetylcholinesterase and butyrylcholinesterase enzymes. For acetylcholinesterase (AChE) inhibitory assay, 140 lL of 0.1 M sodium phosphate buffer of pH 8 was first added to a 96-wells microplate followed by 20 lL

of test samples and 20 lL of 0.09 units/mL acetylcholinesterase enzyme. After 15 min. of incubation at 25 °C, 10 lL of 10 mM 5,50 -dithiobis-2-nitrobenzoic acid (DTNB) was added into each well followed by 10 lL of 14 mM acetylthiocholine iodide. Thirty minutes after the initiation of enzymatic reaction, absorbance of the colored end-product was measured using BioTek Power Wave X 340 Microplate Spectrophotometer at 412 nm. For butyrylcholinesterase (BuChE) inhibitory assay, the same procedure described above was followed, except for the use of enzyme and substrate, instead of which, butyrylcholine esterase from equine serum and S-butyrylthiocholine chloride were used. IC50 value was calculated by standard protocol [4-5]
Each test was conducted in triplicate. Absorbance of the test samples was corrected by subtracting the absorbance of their respective blank. Percentage inhibition was calculated using the following formula:

$$\text{Percentage of inhibition} = \frac{\text{Absorbance of control} - \text{Absorbance of Sample}}{\text{Absorbance of control}} \times 100$$

Selectivity for AChE defined as IC_{50}(BChE)/IC_{50}(AChE).
Selectivity for BChE defined as IC_{50}(AChE)/IC_{50}(BChE).

References

1. T. Ahmed, A.H. Gilani. Inhibitory effect of curcuminoids on acetylcholinesterase activity and attenuation of scopolamine-induced amnesia may explain medicinal use of turmeric in Alzheimer's disease. *Pharmacol Biochem Behav*. 2009, 91(4):554-9..
2. Y. kia, H. Osman, R.Suresh Kumar, A. Basiri, V. Murugaiyah Bioorg. Med. Chem. 2014, 22, 1318-1328
3. A. Basiri, B. M. Abd Razik, M. Oday Ezzat, Y. Kia, R. Suresh Kumar, A. I. Almansour, N. Arumugam, V. Murugaiyah, Bioorg. Chem. 2017, 75, 210–216
4. B.M. Abd Razik, H. Osman, A. Basiri, A. Salhin, Y. Kia, M.O. Ezzat, V. Murugaiyah. Ionic liquid mediated synthesis and molecular docking study of novel aromatic embedded Schiff bases as potent cholinesterase inhibitors. Bioorg Chem. 2014 Dec;57:162-168.
5. A. Basiri, V. Murugaiyah, H. Osman, R.S. Kumar, Y. Kia, K.B. Awang, M.A. Ali. An expedient, ionic liquid mediated multi-component synthesis of novel piperidone grafted cholinesterase enzymes inhibitors and their molecular modeling study. Eur J Med Chem. 2013 Sep;67:221-9.

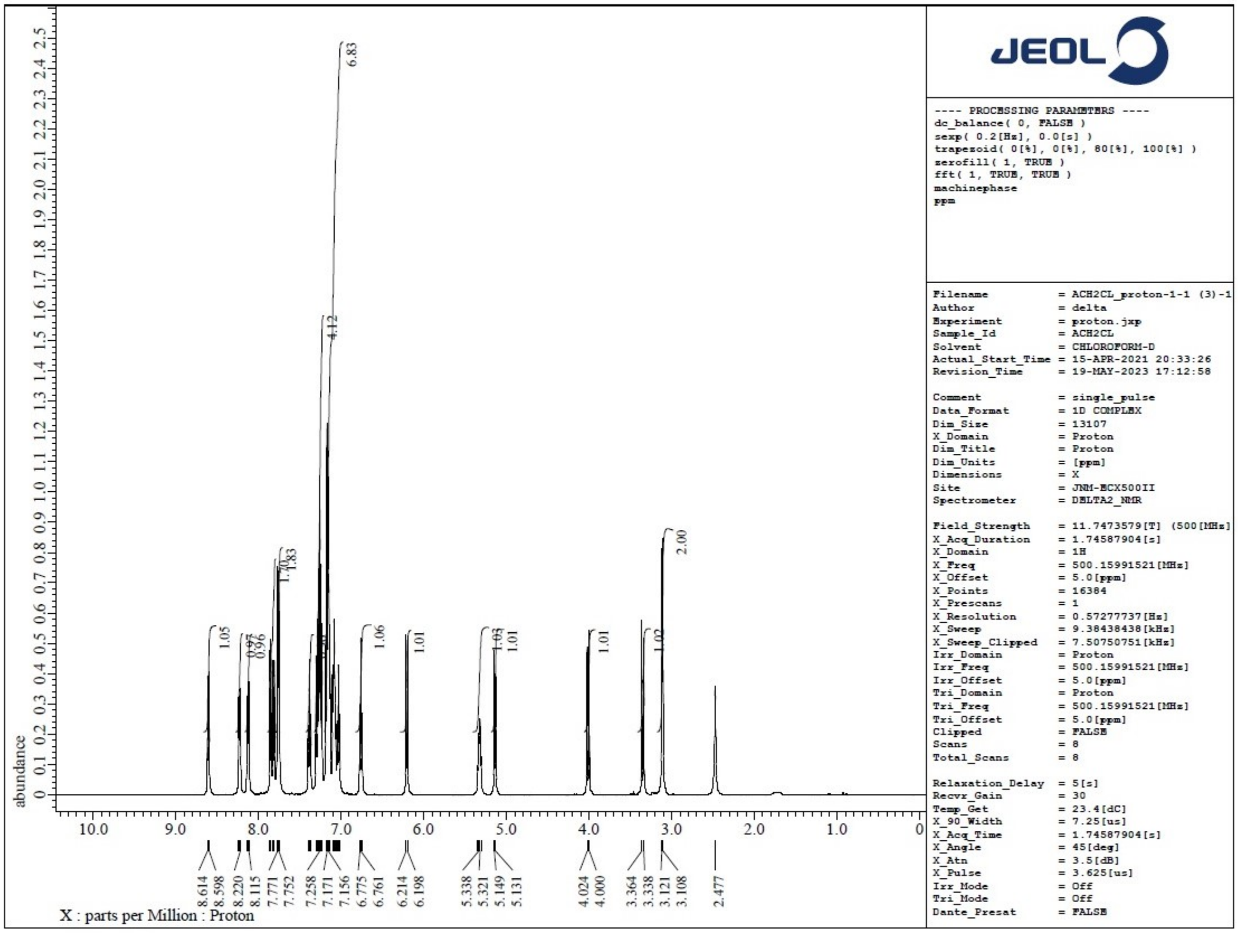

Figure S1. ^{1}H NMR spectrum of **5c**

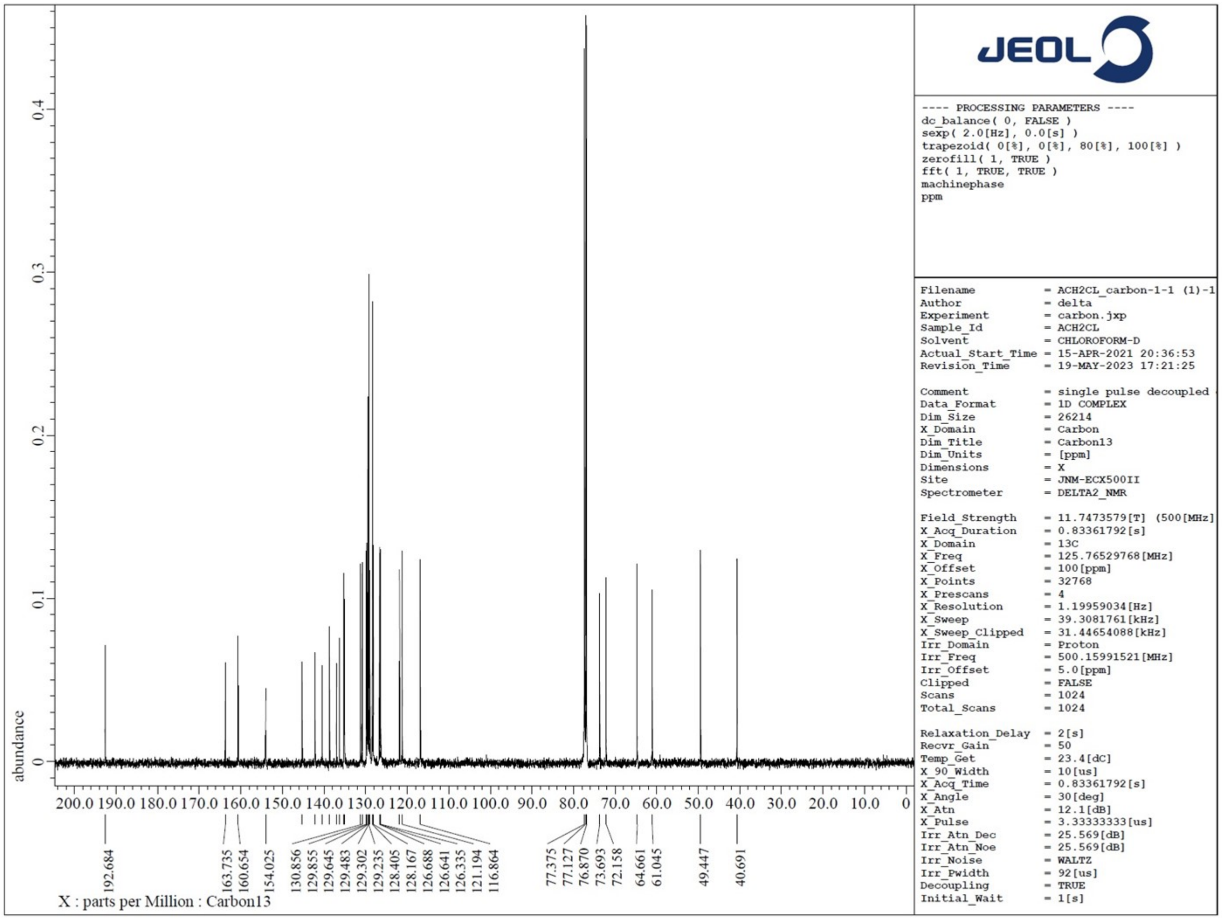

Figure S2. ^{13}C NMR spectrum of **5c**

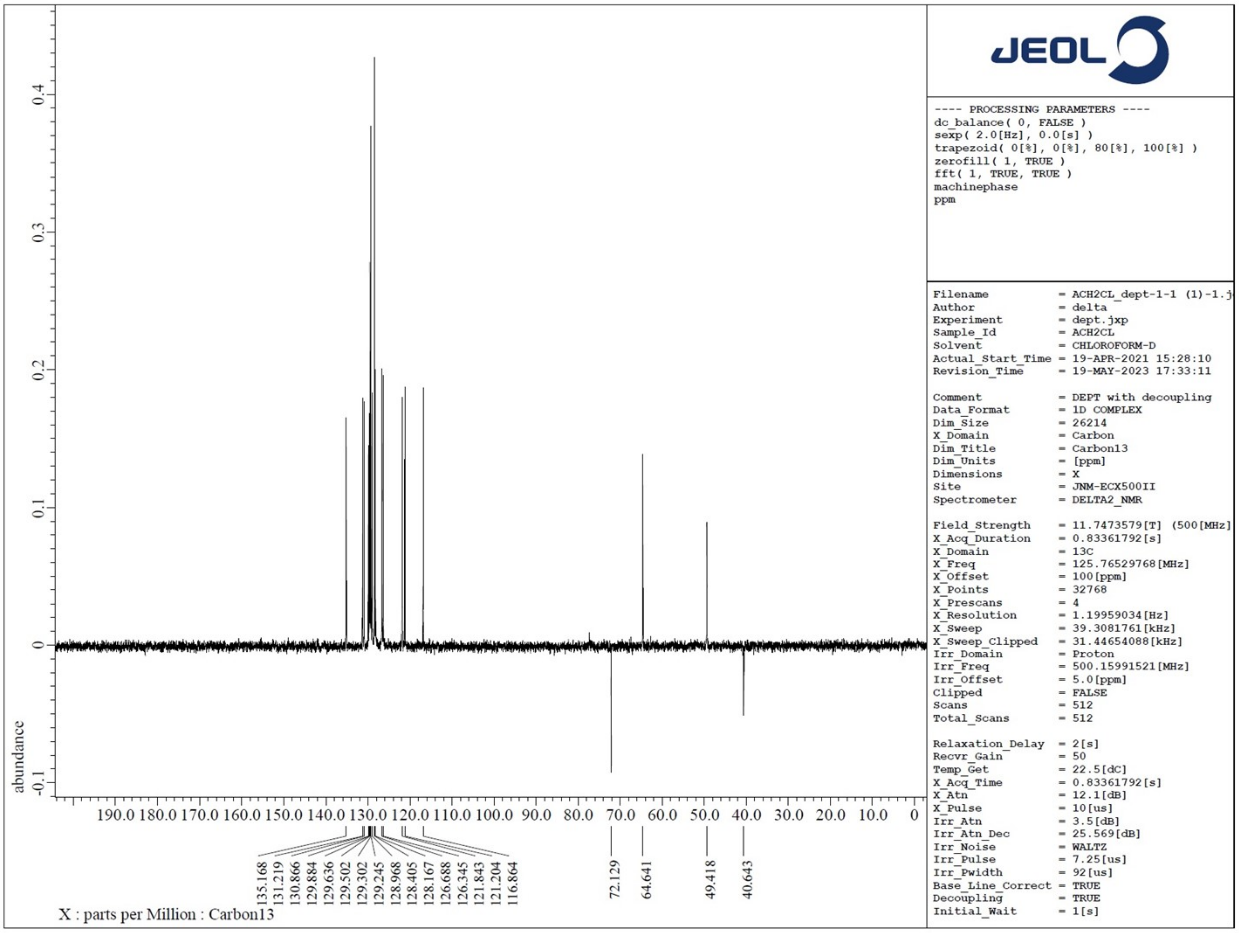

Figure S3. DEPT-135 spectrum of **5c**

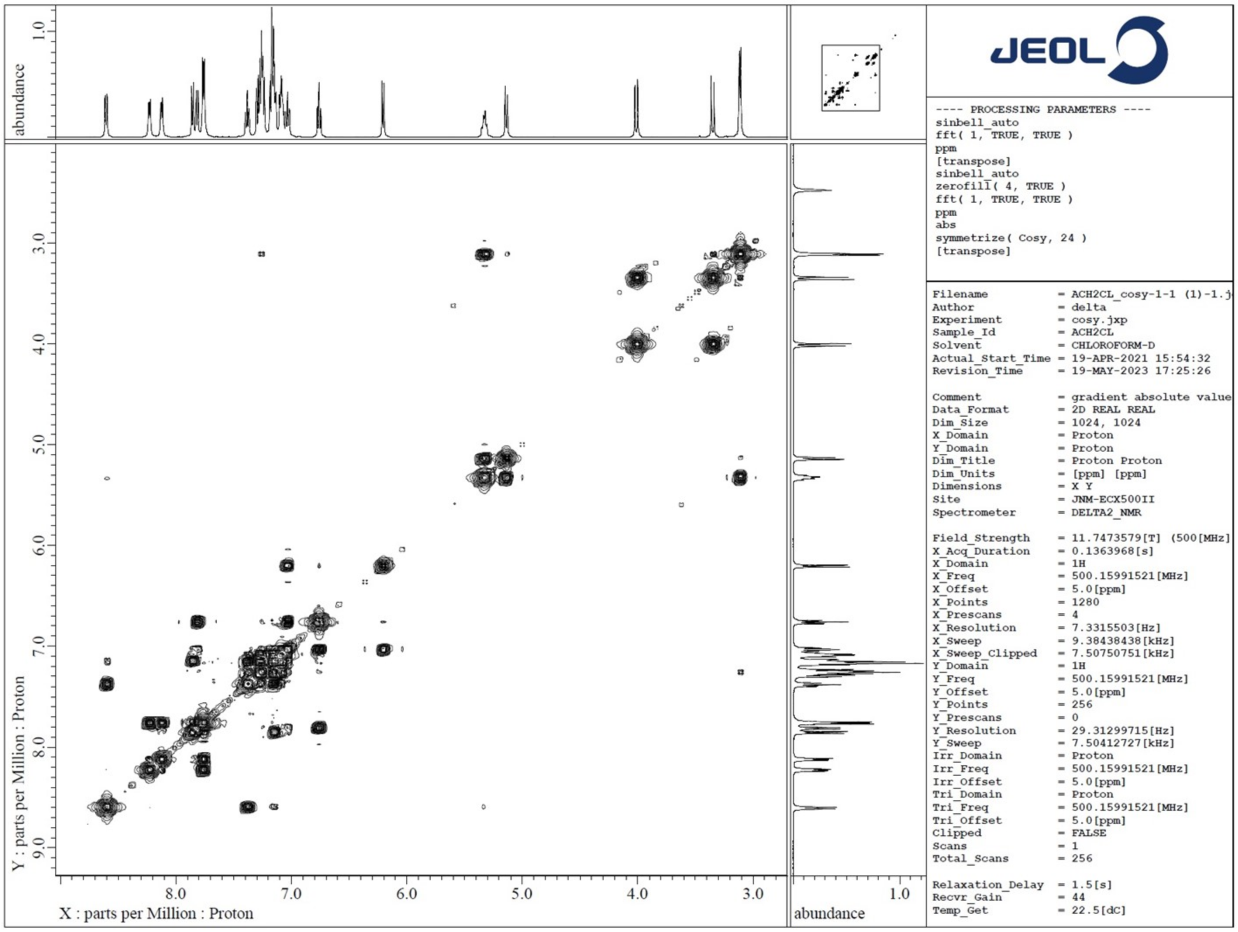

Figure S4. ^{1}H, ^{1}H-COSY spectrum of **5c**

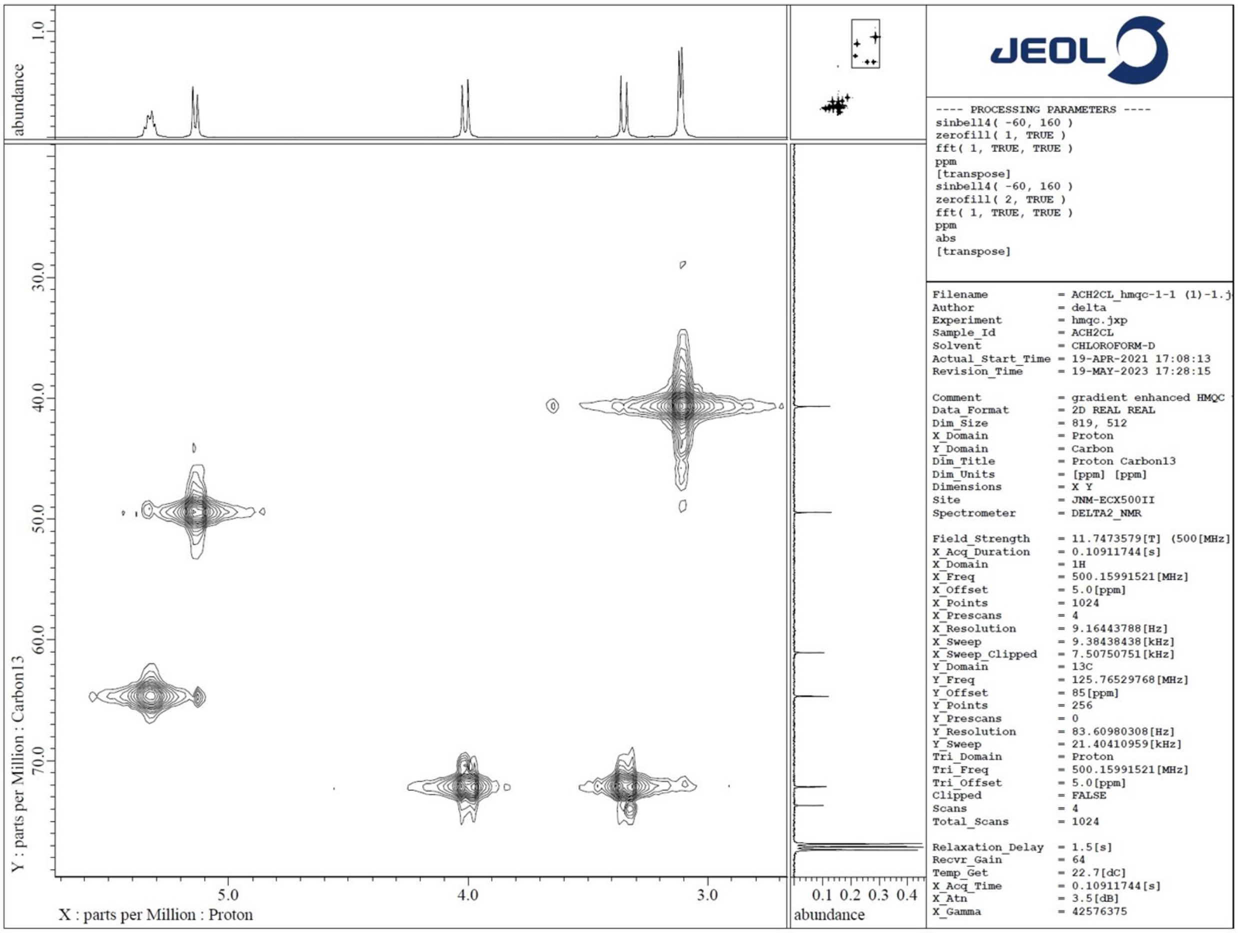

Figure S5. HMQC NMR spectrum of **5c**

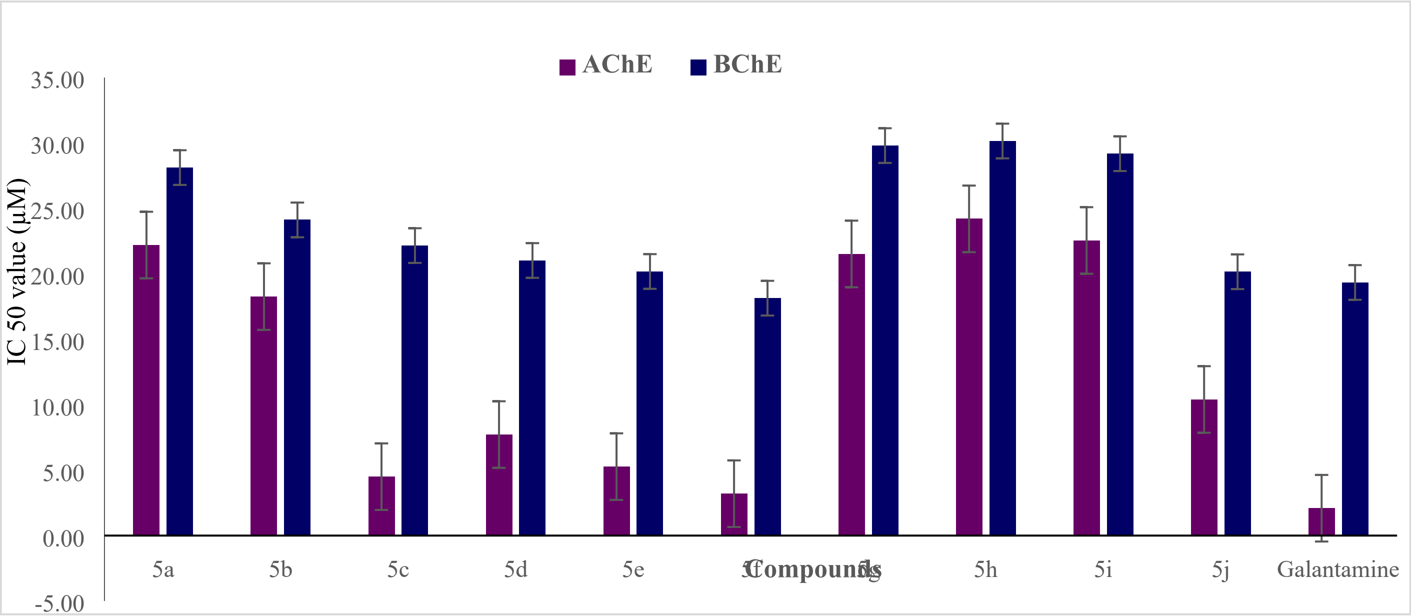

Figure S6. Cholinesterase inhibitory activity

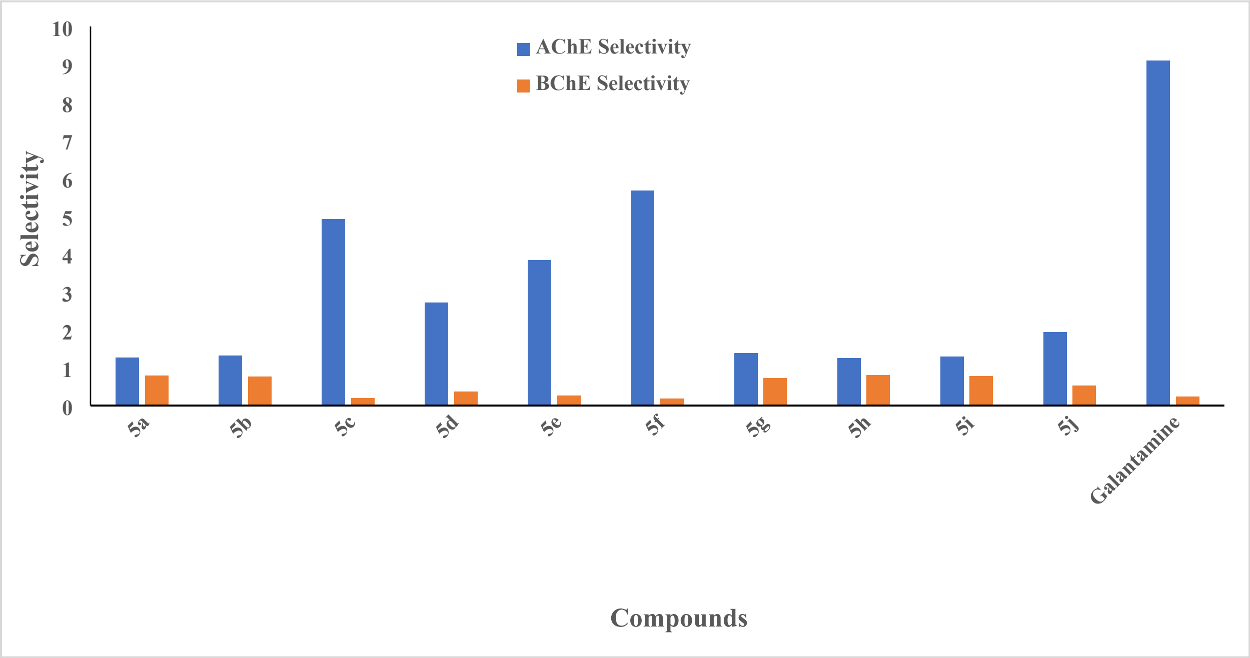

Figure S7. Cholinesterase seletivity

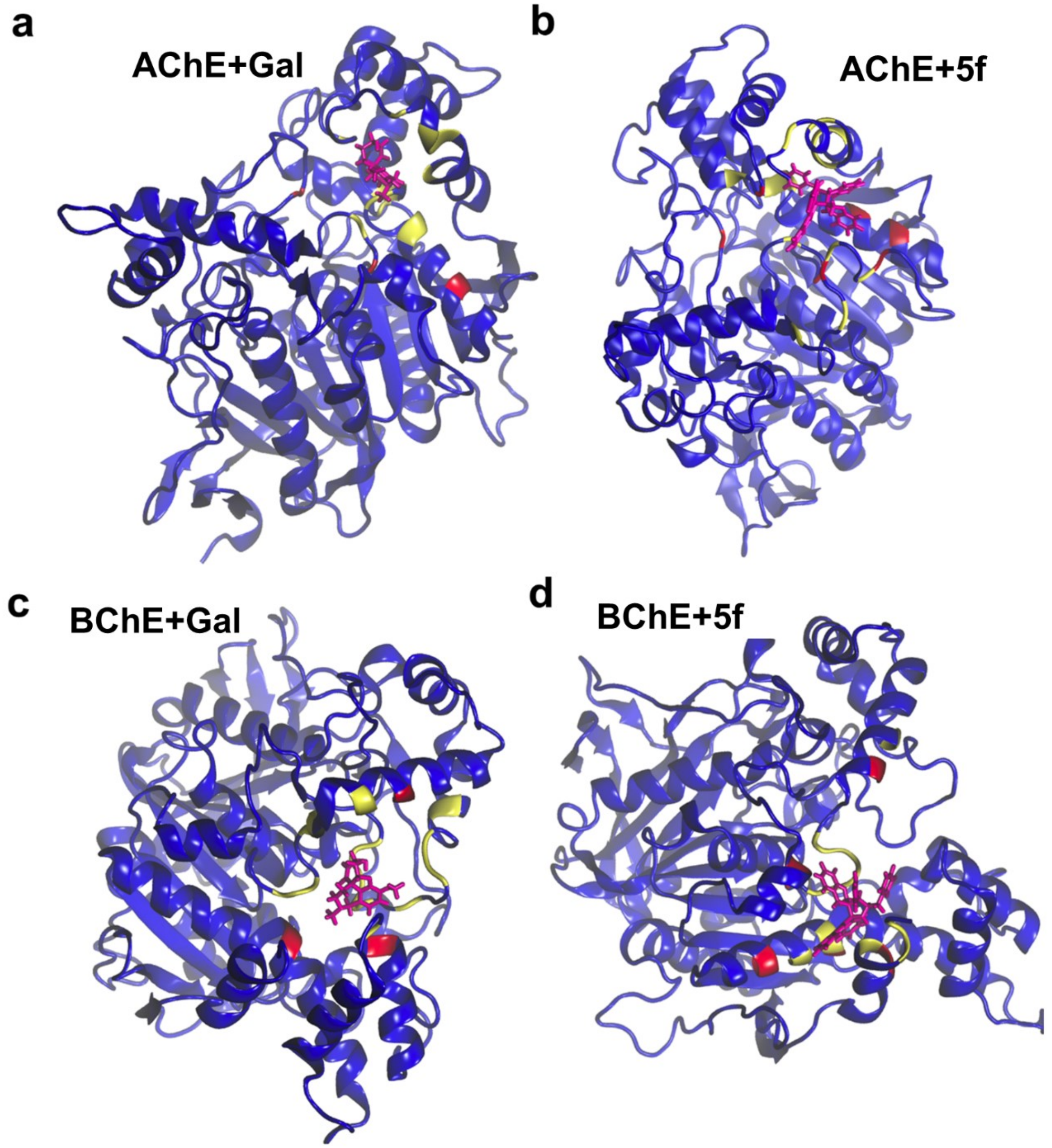

Figure S8: Representation of binding residues (negative/positive binding energy residues represented as Yellow/Red respectively) that are contributing to drug (represented as Magenta) binding with protein (represented as Blue) in (a) AChE+Gal, (b) AChE+**5f**, (c) BChE+Gal and (d) BChE+**5f** complexes. A smaller number of binding residues are observed at galantamine – AChE interface compared to other drug – protein complexes.

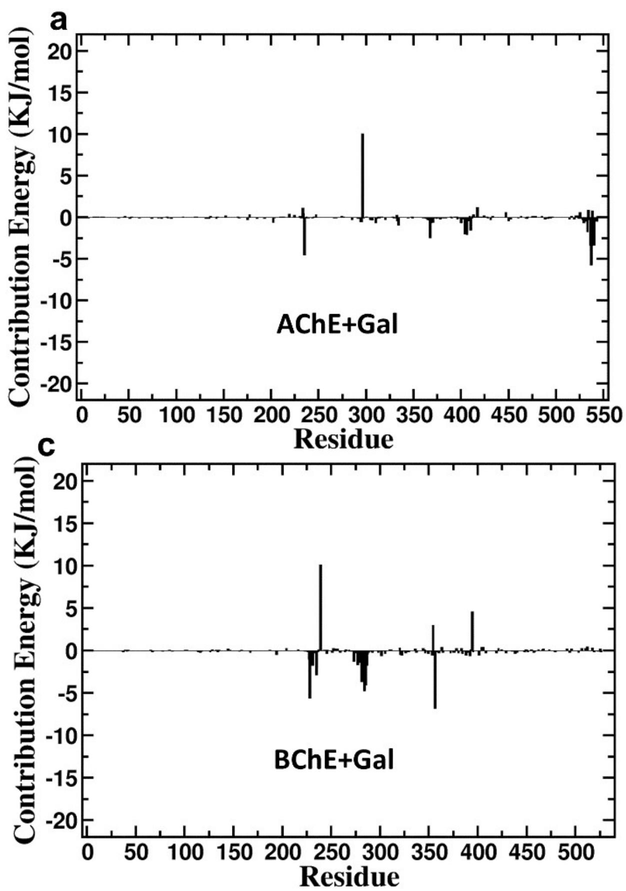

Figure S9: Binding energy profiles of (a) Gal+ AChE and (b) **5f**+AChE, (c) Gal+BChE and (d) **5f**+BChE complexes as a function of its residue number are obtained using MMPBSA calculations. The number of residues contributing to the protein – drug complex formation is computed to explain the differences in their binding energies.

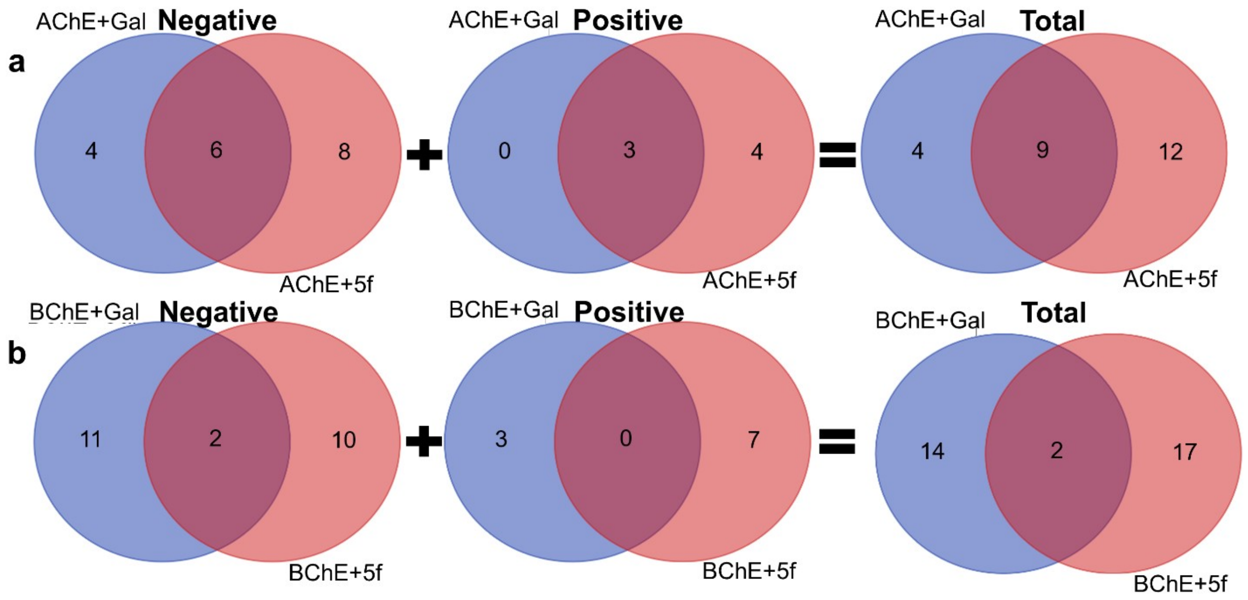

Figure S10: Venn diagrams of number of residues of proteins (a) AChE and (b) BChE that are contributing to protein – drug (Galantamine/**5f**) complex formation. It reveals that the number of residues that favor Galantamine/**5f** binding with the AChE are 10 & 14 respectively, while it changes to 13 & 12 for BChE+Gal and BChE+**5f** complexes. It is in good agreement with the van-der-Waal (vdW) energies observed in protein – ligand complexes.

Table S1: Swiss ADME results for 5f and galantamine.

Swiss ADME results		
	**5f**	**Galantamine**
**Pharmacokinetics**		
GI absorption	Low	High
BBB permeant	No	Yes
P -gp substrate	No	Yes
CYP1A2 inhibitor	Yes	No
CYP2C19 inhibitor	No	No
CYP2C9 inhibitor	No	No
CYP2D6 inhibitor	No	Yes
CYP3A4 inhibitor	No	No
Log K_p (skin permeation)	-5.27 cm/s	-6.75 cm/s
**Physicochemical Properties**		
Molecular weight	589.66 g/mol	287.35 g/mol
Num. heavy atoms	45	21
Num. arom. heavy atoms	34	6
Fraction Csp3	0.15	0.53
Num. rotatable bonds	3	1
Num. H-bond acceptors	6	4
Num. H-bond donors	1	1
Molar Refractivity	174.46	84.05
**Drug likeness**		
Lipinski	No; 2 violations: MW>500, MLOGP>4.15	Yes; 0 violation
Ghose	No; 4 violations: MW>480, WLOGP>5.6, MR>130, #atoms>70	Yes
Veber	Yes	Yes
Egan	No; 1 violation: WLOGP>5.88	Yes
Muegge	No; 2 violations: XLOGP3>5, #rings>7	Yes
Bioavailability	0.17	0.55
**Lipophilicity**		
Log $P_{0/w}$ (iLOGP)	4.38	2.66
Log $P_{0/w}$ (XLOGO3)	6.52	1.84
Log $P_{0/w}$ (WLOGP)	7.18	1.32
Log $P_{0/w}$ (MLOGP)	4.88	1.74
Log $P_{0/w}$ (SILICOS-IT)	7.80	2.03
Consensus Log $P_{0/w}$	6.15	1.92
**Water Solubility**		
Log S (ESOL)	-7.96	-2.93
Solubility	6.40e-06 mg/ml; 1.08e-08 mol/l	3.41e-01 mg/ml; 1.19e-03 mol/l
Class	Poorly soluble	Soluble
Log S (Ali)	-7.66	-2.34
Solubility	1.28e-05 mg/ml; 2.17e-08 mol/l	1.31e+00 mg/ml; 4.56e-03 mol/l
Class	Poorly soluble	Soluble
Log *S* (SILICOS-IT)	-14.64	-2.96
Solubility	1.35e-12 mg/ml; 2.29e-15 mol/l	3.17e-01 mg/ml; 1.10e-03 mol/l
Class	Insoluble	Soluble
